# Fuchs Endothelial Corneal Dystrophy Associations with Systemic Disease, Lifestyle, and Nutritional Intake

**DOI:** 10.1016/j.xops.2025.100899

**Published:** 2025-07-31

**Authors:** Myriam Böhm, Aaron R. Kaufman, Francesca Kahale, Pia Leon, Viridiana Kocaba, Ula V. Jurkunas

**Affiliations:** 1 Cornea Service, Department of Ophthalmology, Massachusetts Eye and Ear Infirmary, Harvard Medical School, Boston, Massachusetts; 2 Schepens Eye Research Institute of Massachusetts Eye and Ear, Harvard Medical School, Boston, Massachusetts; 3 Department of Ophthalmology, Goethe University Clinic, Frankfurt am Main, Germany; 4 Tissue Engineering and Cell Therapy Group, Singapore Eye Research Institute, Singapore

**Keywords:** Comorbidities, Diet and nutrition, Fuchs endothelial corneal dystrophy, Lifestyle risk factors, Smoking

## Abstract

**Objective:**

To evaluate associations between Fuchs endothelial corneal dystrophy (FECD) and systemic comorbidities, lifestyle factors, and dietary patterns, aiming to identify modifiable risk factors and inform therapeutic strategies.

**Design:**

Case-control study integrating a cross-sectional survey with a retrospective chart review conducted at a tertiary referral center.

**Subjects:**

The cohort included 100 individuals from the Cornea Service at Massachusetts Eye and Ear Infirmary: 50 patients with FECD and 50 age- and sex-matched controls within a 10-year range. Four controls were excluded due to protocol adherence.

**Methods:**

Data collection involved chart reviews and a validated 152-item semiquantitative food frequency questionnaire. Smoking and exercise behaviors were assessed through structured questionnaires. Nutrient intake was adjusted for energy using the residual method. Thus, the energy-adjusted intake estimate is the residual from a regression model in which total energy intake is the independent variable and absolute nutrient intake is the dependent variable. Statistical comparisons included Wilcoxon rank-sum and Fisher exact tests (*P* < 0.05).

**Main Outcome Measures:**

Prevalence and statistical significance of systemic comorbidities, lifestyle behaviors, and dietary intake associated with FECD.

**Results:**

Fuchs endothelial corneal dystrophy was associated with hyperlipidemia (74% vs. 50%, *P* = 0.023) and atrial fibrillation (26% vs. 8%, *P* = 0.031). Cumulative tobacco exposure was higher in patients with FECD (11.2 ± 15.1 vs. 6.1 ± 14.1 pack-years, *P* = 0.017). Patients with FECD had higher caloric intake (1862 ± 673 vs. 1463 ± 584 kcal, *P* = 0.027), reduced total fat (*P* = 0.036) and monounsaturated fat (*P* = 0.024), and increased sodium intake (*P* = 0.021). Elevated intake of zinc (*P* = 0.011), manganese (*P* = 0.043), and selenium (*P* = 0.007) was also observed.

**Conclusions:**

Fuchs endothelial corneal dystrophy exhibits significant associations with cardiovascular comorbidities, cumulative tobacco exposure, and specific nutritional factors. The identified associations suggest possible directions for future intervention studies as on cardiovascular management, smoking cessation, and dietary modification. Further studies are warranted to explore mechanistic links and develop preventive and therapeutic strategies.

**Financial Disclosure(s):**

Proprietary or commercial disclosure may be found in the Footnotes and Disclosures at the end of this article.

Fuchs endothelial corneal dystrophy (FECD) is a degenerative corneal disease characterized by endothelial dysfunction, marked by a decreased endothelial cell density, the presence of guttae, and the loss of endothelial barrier and pump function.^1^ The pathophysiology of FECD is multifactorial, involving both genetic predisposition and environmental influences.^1^ Factors such as oxidative stress, estrogen, and ultraviolet light have been linked to FECD pathogenesis.^1^ Research into the underlying mechanisms of FECD continues to evolve.

Although the phenotype of FECD is defined based on ophthalmic clinical findings, it is increasingly recognized that systemic health factors may influence its pathophysiology.^1^, ^2^, ^3^, ^4^, ^5^, ^6^, ^7^ Systemic conditions, such as diabetes or hypertension, are known risk factors for ophthalmic disease, while ophthalmic diagnoses can uncover underlying systemic conditions.^2^ In FECD, systemic associations with cardiovascular disease,^5^ diabetes mellitus,^4^^,^^6^ and smoking^4^^,^^7^ have been reported.

Additionally, FECD is more prevalent in women, with estrogen-dependent oxidative stress pathways proposed as a contributing factor.^8^^,^^9^ Despite these insights, the relationship between systemic health and FECD remains incompletely defined. Notably, the role of nutrition in the development and progression of FECD has been largely underexplored. As nutrition influences systemic inflammation and oxidative stress—key drivers of endothelial dysfunction—it may also contribute to FECD pathogenesis. Investigating dietary patterns and nutrient intake could provide critical insights into modifiable risk factors and potential therapeutic strategies.

The current study aims to address these gaps by examining associations between FECD and systemic comorbidities, lifestyle factors, and dietary intake. By integrating data from nutritional and lifestyle surveys with medical chart reviews, this study seeks to identify actionable risk factors and provides a foundation for future research into the systemic context of FECD.

## Methods

### Study Design and Recruitment

This case-control study combined a cross-sectional survey alongside a retrospective chart review. Patients with FECD were recruited from the Cornea Service and control patients from the Comprehensive and Cataracts Eye Service at the Massachusetts Eye and Ear Infirmary during routine new or follow-up visits to the clinic. Informed consent was obtained from all participants. The inclusion criteria for the group with FECD were clinical diagnosis of FECD. The control group comprised individuals without FECD and no guttae on slit lamp examination, matched to participants with FECD by age (within 10 years) and sex. A total of 104 patients were included, and 4 patients (2 cases and 2 controls) were excluded because the questionnaire was not returned. A total of 100 patients were analyzed (50 case and 50 control); 4 control patients were excluded due to the case-control matching protocol. Paper-based questionnaires were completed in person by participants. This study was approved by the Institutional Review Board at the Massachusetts Eye and Ear Infirmary and it adhered to the Declaration of Helsinki and the Health Insurance Portability and Accountability Act.

### Chart Review

Medical chart review collected data on demographics (age, race, sex, height, and weight), medical history, and FECD stage using a modified Krachmer scale.^10^^,^^11^ Clinical grading of FECD was performed at the slit lamp according to the following guidelines: grade 1—nonconfluent guttae; grade 2—presence of any area of confluent guttae, but without edema or clinical thickening; grade 3—confluent guttae with edema or clinical thickening; and grade 4—edema associated with whitening or haze.^11^

Body mass index in kg/m^2^ was calculated from weight and height. However, it could not be determined for 5 patients with FECD and 2 controls due to incomplete data.

### Nutritional and Lifestyle Questionnaires

Nutritional intake was assessed using a validated 152-food-item semiquantitative food frequency questionnaire (SFFQ) (Supplemental Appendix 1, available at www.ophthalmologyscience.org).^12^ The SFFQ queried the frequency of consumption of various foods, beverages, and vitamin or supplements consumed over the past year. Nutrient intakes were calculated from the questionnaire by multiplying a weight proportional to the intake frequency by the nutrient composition for the portion size specified for each food or vitamin supplement. Nutrients were then summed across all foods and supplements to obtain the total intake for each individual. Also, intakes not including supplements were calculated.^12^

Survey responses were processed by the Nutrition Questionnaire Service Center at the Harvard School of Public Health where daily intake of individual nutritional factors was computed by assigning a daily frequency weight using an internal database.

For nutrition analysis, an energy-adjustment of nutrition factors was performed using the residual method.^13^, ^14^, ^15^ Energy-adjusted intakes are relevant because individuals alter their intakes of specific nutrients by changing the composition of their diet, keeping total energy constant. Total energy intake can confound associations with specific nutrients if any of these factors are associated with disease risk. Thus, adjustment for total energy intake is important to control for confounding, reduce extraneous variation, and predict the effect of dietary interventions.

There exist disease-risk models, and formulations of these models are available to account for energy intake in epidemiologic analyses, including adjustment of nutrient intakes for total energy intake by regression analysis (residual model) and addition of total energy to a model with the nutrient density (nutrient density model: nutrient divided by energy).

The nutrient-residual (energy-adjusted) model, however, offers the advantage that it can be used as an alternative to using the nutrient densities model to control for confounding by total energy intake and to remove extraneous variation due to energy intake. The residual model uses regression analyses to compute residuals of nutrient intake by removing the variation caused by total energy intake.^15^ In the nutrient-residual approach, the energy-adjusted intake estimate is the residual from the regression model in which total energy intake (reference energy level of 1800 kcal) is the independent variable and absolute nutrient intake is the dependent variable. The residual is thus an estimate of nutrient intake uncorrelated with total energy intake that is directly related to overall variation in food choice and composition of an individual.

Exercise and smoking behavior were assessed by a lifestyle questionnaire (Appendix 2, available at www.ophthalmologyscience.org). A cigarette pack size of 20 cigarettes was presumed. Cumulative burden exposure of pack-years was calculated from reported smoking duration and daily pack consumption.

### Statistical Methods

Statistical analysis was performed using Stata standard edition version 17.0 (StataCorp LLC). Descriptive statistics, including mean and standard deviation, were calculated for continuous variables. The Wilcoxon rank-sum test was used for comparisons of continuous variables, and Fisher exact test was used for categorical variables between FECD and control groups. Statistical significance was set as *P* < 0.05.

## Results

### Demographics

The mean age was 67.7 ± 9.6 in the FECD group and 68.3 ± 9.2 years in the control group (*P* = 1.000). In both groups, 64% of patients were female (*P* = 1.000). Within the FECD group, 38% of patients had stage 3 or greater FECD. In total, 13 of the 50 patients with FECD (30%) had undergone keratoplasty, and 3 had undergone keratoplasty in both eyes (endothelial keratoplasty: 13, penetrating keratoplasty: 3). The mean body mass index was 26.5 ± 5.5 kg/m^2^ in FECD cases and 26.2 ± 4.9 kg/m^2^ in control cases (*P* = 0.879) (Table 1).

**Table 1:** Demographic Characteristics, FECD Stage, and Comorbidity

	FECD Cases	Control Cases	*P* Value
Patients (n)	50	50	
Age, mean ± SD (range), yr	67.7 ± 9.6 (49–90)	68.3 ± 9.2 (47–82)	1.000
Female sex, n (%)	32 (64%)	32 (64.0%)	1.000
Race			
White, n (%)	50 (100%)	48 (96%)	0.495
Black, n (%)	0 (0%)	0 (0%)	
Indian, n (%)	0 (0%)	1 (2%)	
Asian, n (%)	0 (0%)	1 (2%)	
FECD-modified Krachmer stage, mean ± SD (range)	2.7 ± 1.2 (1–4)	n/a	
Stage 1, n (%)	9 (18%)	n/a	
Stage 2, n (%)	17 (34%)	n/a	
Stage 3, n (%)	19 (38%)	n/a	
Stage 4, n (%)	5 (10%)	n/a	
Comorbidity			
Hypertension	34 (68%)	25 (50%)	0.103
Hyperlipidemia	37 (74%)	25 (50%)	**0.023**
Coronary artery disease	10 (20%)	4 (8%)	0.148
Congestive heart failure	6 (12%)	1 (2%)	0.112
Atrial fibrillation	13 (26%)	4 (8%)	**0.031**
Peripheral vascular disease	3 (6%)	0 (0%)	0.242
Aortic stenosis	5 (10%)	1 (2%)	0.204
Carotid disease	1 (2%)	1 (2%)	1.000
Diabetes mellitus, type 2	5 (10%)	5 (10%)	1.000
History of stroke	5 (10%)	1 (2%)	0.204
BMI, kg/m2, mean ± SD	26.5 ± 5.5∗	26.2 ± 4.9∗	0.879
Dietary supplement use (yes), n (%)	33 (66%)	21 (42%)	**0.027**

BMI = body mass index; FECD = Fuchs endothelial corneal dystrophy; n/a = not applicable; SD = standard deviation.

Bolded *P* values denote statistical significance (*P* < 0.05).

∗ BMI was calculated for 45 patients in the FECD group and 48 in the control group, as height and weight for BMI computation were not available in the remaining patients.

### Comorbidity

Cardiovascular disease occurred frequently in both groups, but associations with FECD were observed. Significant differences were found for hyperlipidemia (74% FECD and 50% controls, *P* = 0.023) and atrial fibrillation (26% FECD and 8% controls, *P* = 0.031). A trend toward significance was observed in other cardiovascular diseases: hypertension (68% FECD and 50% controls, *P* = 0.103), congestive heart failure (12% FECD and 2% controls, *P* = 0.112), and coronary artery disease (20% FECD and 8% controls, *P* = 0.148). No statistically significant associations were observed for other comorbid diseases (Table 1).

### Lifestyle Questionnaire

Smoking and exercise behavior are summarized in Table 2. The proportion of patients who had ever smoked was greater in the FECD group than in the control group (56% vs. 38%), but this difference was not significant (*P* = 0.109). For the subsets of patients who had ever smoked, there were no differences between the FECD and control groups for age starting smoking (*P* = 0.260), daily smoking behavior (*P* = 0.282), and duration of smoking (*P* = 0.625). However, the mean cumulative tobacco exposure across all patients in the 2 groups was significantly greater in the FECD group than in the control group (11.2 ± 15.1 vs. 6.1 ± 14.1 pack-years, *P*=0.017). For the subset of patients who had ever smoked, there was no statistically significant difference in the mean cumulative tobacco exposure (20.8 ± 14.9 vs. 19.1 ± 19.1 pack-years, *P* = 0.131).

**Table 2:** Smoking and Exercise Behavior

Smoking Behavior	FECD Cases	Control Cases	*P* Value
Ever smoked tobacco, yes, n (%)	28 (56%)	19 (38%)	0.109
Age started smoking (for current smokers), mean ± SD (range), yrs	18.2 ± 6.0 (9–46)	17.0 ± 4.0 (12–30)	0.260
Daily smoking behavior for ever smoked, mean ± SD (range), pack/day	0.83 ± 0.46 (0.15–2.0)	0.72 ± 0.45 (0.1–2.0)	0.282
Duration of smoking for ever smoked, mean ± SD (range), yrs	24.1 ± 12.1 (2–50)	23.7 ± 16.4 (3–62)	0.625
Cumulative tobacco exposure, mean ± SD (range), pack-yrs			
All patients	11.2 ± 15.1 (0–60)	6.1 ± 14.1 (0–63)	**0.017**
Subgroup of patients who ever smoked	20.8 ± 14.9 (0.3–60)	19.1 ± 19.1 (1.5–63)	0.131
Weekly duration of exercise, mean ± SD (range), hrs/wk	4.7 ± 3.9	4.6 ± 3.2	0.840

FECD = Fuchs endothelial corneal dystrophy; SD = standard deviation.

Bolded *P* value denotes statistical significance (*P* < 0.05).

There was no significant difference in the mean weekly duration of exercise between FECD or control patients (4.7 ± 3.9 vs. 4.6 ± 3.2 h/week, *P* = 0.840).

### Nutritional Factors

A focused list of nutritional factors from the SFFQ is included in Table 3; the full list of nutritional factors computed by the SFFQ can be found in Appendix 3 (available at www.ophthalmologyscience.org).

**Table 3:** Absolute Daily Nutrient Intakes of the Study Population Estimated by Semiquantitative Food Frequency Questionnaire

Nutrient or Dietary Constituent (Selected Items)	Daily Intake, to 1800 kcal Daily in (Adjusted Take), Mean ± SD		*P* Value
	FECD	Control	
Total energy, kcal	1862 ± 673	1463 ± 584	**0.027**
Protein, g	76.2 ± 16.6	73.5 ± 15.3	0.345
Fat and lipid			
Total fat, g	64.8 ± 14.9	71.0 ± 15.3	**0.036**
Saturated fat, g	20.9 ± 5.0	21.6 ± 5.6	0.612
Monounsaturated fat, g	23.9 ± 8.1	28.1 ± 10.7	**0.024**
Polyunsaturated fat, g	14.5 ± 3.3	15.7 ± 4.4	0.224
Total trans fat, g	0.93 ± 0.25	0.90 ± 0.36	0.240
Total omega 3, g	1.88 ± 0.67	2.10 ± 0.86	0.288
Cholesterol, mg	239.2 ± 109.3	234.8 ± 103.9	0.896
Carbohydrates			
Carbohydrate, g	220.8 ± 42.7	210.7 ± 36.9	0.253
Whole grain amount, g	42.2 ± 27.5	32.6 ± 21.8	**0.031**
Insulinogenic load, glucose	711.7 ± 119.8	667.8 ± 135.1	0.077
Dietary minerals			
Sodium, mg	2036.1 ± 436.2	1833.7 ± 402.2	**0.021**
Potassium, mg	3111.7 ± 559.2	3152.6 ± 518.2	0.659
Calcium, mg	1323.2 ± 628.4	1154.0 ± 583.8	0.104
Iron, mg	17.2 ± 10.6	15.8 ± 13.5	0.136
Magnesium, mg	382.6 ± 105.2	392.8 ± 135.4	0.951
Phosphorus, mg	1320.9 ± 209.8	1272.3 ± 274.1	0.206
Zinc, mg	23.5 ± 22.6	16.9 ± 19.2	**0.011**
Manganese, μg	5.11 ± 1.88	4.42 ± 2.26	**0.043**
Selenium, μm	16.5 ± 22.9	10.5 ± 24.1	**0.007**
Copper, mg	2.04 ± 1.34	1.77 ± 1.11	0.211
Vitamins			
Thiamine, mg	5.34 ± 12.41	8.54 ± 18.15	0.988
Riboflavin, mg	6.14 ± 12.46	9.40 ± 18.20	0.772
Niacin, mg	55.9 ± 96.8	68.7 ± 139.3	0.697
Pantothenic acid, mg	15.1 ± 14.9	16.7 ± 20.2	0.558
Pyridoxine, mg	17.6 ± 47.5	18.0 ± 35.4	0.978
Total folate 1998 μg	730.3 ± 356.4	641.6 ± 391.8	0.098
Cobalamin, μg	146.3 ± 304.4	97.0 ± 243.7	0.181
Vitamin C, mg	301.6 ± 352.0	250.3 ± 325.1	0.281
Vitamin D, IU	1413.1 ± 1183.7	1301.2 ± 1060.4	0.540
Vitamin C, μg	114.2 ± 57.2	113.8 ± 52.8	0.768
Vitamin E, mg	23.3 ± 25.9	19.5 ± 14.6	0.222
Vitamin K1, μg	196.0 ± 161.8	215.1 ± 144.3	0.478
Carotenoids			
β-Carotene, μg	7021.8 ± 6227.6	7751.8 ± 5704.0	0.823
β-Cryptoxanthin, μg	230.2 ± 207.6	233.2 ± 144.3	0.628
Lycopene, μg	4621.3 ± 3439.1	4578.2 ± 4246.6	0.622
Lutein-zeaxanthin, μg	3894.3 ± 3124.5	4282.1 ± 3391.1	0.595

FECD = Fuchs endothelial corneal dystrophy; IU = international units; SD = standard deviation.

Bolded *P* value denotes statistical significance (*P* < 0.05).

Daily caloric intake was higher in patients with FECD than in control (1862 ± 673 vs. 1463 ± 584 kcal, *P* = 0.027). However, when nutritional factors were calculated using the residual method, patients with FECD had lower intake of total fat (*P* = 0.036) and monosaturated fat (*P* = 0.024). There were no statistically significant differences in the intake of saturated fat (*P* = 0.612), polyunsaturated fat (*P* = 0.224), trans fat (*P* = 0.240), or cholesterol (*P* = 0.896).

There was no difference in daily intake of carbohydrates overall (*P* = 0.253), although patients with FECD had greater intake of whole grains (*P* = 0.031). Insulinogenic load was not statistically different between the 2 groups (*P* = 0.077).

Sodium intake was greater in patients with FECD than in controls (*P* = 0.021). Multiple trace elements had higher intake in patients with FECD: zinc (*P* = 0.011), manganese (*P* = 0.043), and selenium (*P* = 0.007). Moreover, the following elements without supplemental intake were also significantly higher in the FECD compared with the control group: iron without supplements (0.028), zinc without supplements (0.023), and thiamine vitamin B1 without supplements (0.017) (Appendix 3).

## Discussion

This observational study reveals significant associations between FECD and systemic health factors, including higher rates of cardiovascular disease, smoking behavior, elevated sodium (mg) intake, higher whole grain amount (g), and lower total fat (g) and monounsaturated fat (g) amounts.

Additionally, this study pioneers the investigation of nutritional factors in FECD, addressing a critical gap in existing research. These findings underscore the interconnected nature of systemic health and FECD pathophysiology, highlighting the potential for targeted interventions such as cardiovascular risk management, dietary modifications, and smoking cessation to slow disease progression. Furthermore, this work lays the foundation for future studies to explore the mechanistic pathways linking systemic health, nutrition, and FECD, with the goal of advancing both preventive and therapeutic strategies.

In this study, the FECD group exhibited significantly higher rates of cardiovascular diseases, with statistically significant differences observed in hyperlipidemia and atrial fibrillation, as well as trends toward significance in congestive heart failure, hypertension, and coronary artery disease. These findings align with a prior small case-control study,^5^ which also reported an increased prevalence of cardiovascular disease in FECD, though without assessment of specific conditions. Furthermore, Scherer et al^16^ identified a negative correlation between endothelial cell density and mortality from all-cause cardiac disease, including coronary heart disease and hypertension. The increased dietary sodium intake observed in patients with FECD in the current study may further suggest a cardiovascular phenotype, as excessive sodium consumption is a well-established cardiovascular risk factor.^17^ This association may indicate shared mechanisms between endothelial dysfunction in the cornea and systemic vascular health, highlighting the potential role of sodium in exacerbating both ocular and systemic conditions.

Additional studies incorporating physiologic sodium measurements are needed to validate and strengthen this observation.

Interestingly, the study finds a higher caloric intake in patients with FECD, yet lower total fat (g) and monounsaturated fat (g) amount in the FECD group. Contrastingly, the FECD group does not show a consecutively higher carbohydrate or protein intake compared with the control group.

Oleic acid, tea oil, almond oil, and nut oil are commonly used as monounsaturated fatty acids in the daily life of human beings.^18^ In recent years, there has been a debate on the effects of monounsaturated fats on the cardiovascular system. Up till now, contradicting evidence exists whether monounsaturated fatty acids protect from endothelial damage in cardiovascular disease^19^ or not.^20^ A Mediterranean-style diet can be defined by a high monounsaturated/saturated fat ratio. Moreover, a high consumption of whole grains and cereals is a component of a Mediterranean-style diet. Calculating the monounsaturated/saturated ratio for this study finds a higher ratio for the control group (μ:1.38 ± 0.59) compared with the Fuchs group (μ:1.15 ± 0.27), indicating that the control group follows a more Mediterranean-style diet compared with the Fuchs group with respect to fat. On the contrary, the Fuchs group shows a significantly higher whole grain consumption compared with the control group. Despite a relatively large number of studies on Mediterranean-style diet on the prevention of cardiovascular disease, there exists still uncertainty regarding the effects of a Mediterranean-style diet on clinical endpoints and cardiovascular risk factors.^21^

The results of our study reinforce the need for further research to explore the interplay between cardiovascular health, diet, and FECD, with potential implications for patient counseling and risk factor management.

Regarding diabetes, there was no increased occurrence of the condition in patients with FECD in this study; however, a trend toward significance was noted in the daily intake of insulinogenic load of carbohydrates. Diabetes mellitus has been previously linked to morphological, ultrastructural, and functional abnormalities in the endothelium as well as reduced endothelial cell density.^22^^,^^23^ In an observational study, Zwingelberg et al^4^ have reported that diabetes mellitus is associated with more advanced Krachmer stage of FECD. Additionally, an analysis of Veterans Affairs electronic health record data for diagnoses found associations between diabetes mellitus and diabetes-related phecodes.^6^

It is crucial to recognize that the associations observed between FECD and diagnostic comorbidities are influenced by several limitations. Indeed, diagnoses were obtained from the electronic medical record review, and both accuracy or availability of these data may vary. Furthermore, there is potential for selection bias to confound case-control association studies—patients in the case (FECD) and control group may be more likely to have acquired additional nonocular diagnoses by virtue of the participation in both ocular and nonocular health care.

In this comprehensive study, we compared multiple variables between the 2 groups without adjusting for multiple comparisons; therefore, the reported associations should be considered exploratory.

The SFFQ used in this study is validated and has been used in prior studies to associate nutritional parameters in various studies as, for example, in linoleic acid and gluten intake with diabetes disease^24^^,^^25^ and animal and plant protein with cardiovascular disease^26^ among large cohorts from the Nurses' Health Study and the Health Professionals Follow-Up Study. The sample size in this study, with 50 FECD and 50 control patients, is rather small, and further research with a higher sample size and a longer follow-up time is needed to support the observations of this study.

Moreover, the use of minerals and vitamin supplement produces additional complexity, as the nutrients from supplements are typically not correlated with energy intake.^14^

Patients in the FECD case group reported significantly higher supplement use compared with controls, which may reflect counseling during clinical visits encouraging a healthier lifestyle, including vitamin intake. To provide a comprehensive view of nutritional intake, dietary data excluding supplements are also reported (Appendix 3), offering insight into the dietary profiles of both groups with and without supplementation.

This study strengthens the previously established link between smoking and ocular diseases including FECD pathophysiology.^3^^,^^27^, ^28^, ^29^, ^30^, ^31^ Smoking likely contributes to oxidative stress in FECD by increasing free radical production and depleting antioxidants in both the bloodstream and ocular tissues.^1^ This imbalance exacerbates oxidative damage, leading to endothelial cell apoptosis, the formation of corneal guttae, and higher Krachmer grading when adjusted for age.^4^ Moreover, smoking has been associated with endothelial dysfunction in multiple human observational studies^7^^,^^32^^,^^33^ and supported by findings from animal models.^34^ Consistent with these observations, the present study found that mean cumulative tobacco exposure (pack-years) was higher in the FECD group. Additionally, Zwingelberg et al^4^ reported a correlation between smoking and increased Krachmer grade, further aligning with the “Reykjavik Eye Study,” which showed that individuals with a 20 pack-year smoking history face a twofold greater risk of developing corneal guttae.^7^ Prior research on other diseases as, for example, lung cancer or peripheral artery disease,^35^^,^^36^ also find a significant dose-response association between cumulative smoking exposure. The finding of a significantly higher tobacco exposure in the FECD group compared with the control group suggests that the effect of cumulative smoking exposure on FECD seems to be a dose-response relationship. Collectively, these findings reinforce the hypothesis that smoking plays a significant role in the progression and severity of FECD. Given the well-documented oxidative effects of smoking, these results underscore the importance of counseling patients with FECD on smoking cessation as a potential intervention to slow disease progression. Furthermore, these findings highlight the need for future studies to investigate the mechanistic pathways linking smoking to FECD pathogenesis and to explore whether smoking cessation can mitigate endothelial cell loss or improve clinical outcomes in patients with FECD.

The role of nutrition in FECD pathophysiology has been theorized based on parallels to age-related macular degeneration,^1^^,^^37^ which also involves oxidative stress as a key mechanism. In age-related macular degeneration, nutritional factors have been implicated, and nutritional supplements are used to slow disease progression.^37^ In this study, statistically significant differences in antioxidant intake were limited. Unexpectedly, zinc^38^ and manganese^39^—both of which play physiological roles in enzymatic free radical scavenging—had higher intake rates in patients with FECD than in controls. Nevertheless, the conclusions from the nutritional profile are constrained by the single time point of the cross-sectional survey, contrasting with the longitudinal nature of FECD pathogenesis. Moreover, high alcohol consumption has been associated with an increased risk of age-related macular degeneration due to its pro-oxidant effects and disruption of oxidative stress defense.^40^^,^^41^ Hence, the potential impact of alcohol consumption in FECD pathophysiology warrants further investigation, as it was not assessed in this study. Given its detrimental effects in other diseases, including cancer and cardiovascular conditions, future studies should explore its influence on FECD development and progression.

This study lends additional data to emerging understanding of systemic health factors that may be associated with FECD. Based on observations in this study, it may be advisable to counsel patients with FECD on screening for concomitant cardiovascular disease, moderating salt intake to manage cardiovascular risk, and smoking cessation. Further investigations using larger datasets will be essential in refining these recommendations for patient care.
