# Supplementary material for: Fuchs Endothelial Corneal Dystrophy Associations with Systemic Disease, Lifestyle, and Nutritional Intake
Source: Ophthalmol Sci. 2025 Jul 31;6(1):100899. doi: 10.1016/j.xops.2025.100899 (PMC12478083; doi:10.1016/j.xops.2025.100899)
Supplement: Appendix 3 [file mmc1.pdf]

Supplemental Appendix 3: Absolute daily intake of all nutritional factors estimated by semiquantitative food frequency questionnaire

Nutritional factor	Daily Intake, adjusted to 1800 kcal daily intake				
	FECD cases (n = 50 patients)		Control cases (n= 50 patients)		
	Mean	Std. Dev	Mean	Std. Dev	*P*-value
Total energy, kcal	1847.069	673.224	1531.017	583.792	0.027
Protein, g	76.283	16.611	73.478	15.349	0.345
Animal fat, g	27.153	9.220	25.683	10.506	0.262
Vegtable fat, g	37.665	12.279	45.571	17.908	**0.020**
Total fat, g	64.760	14.945	71.006	15.317	**0.036**
Carbohydrate, g	220.840	42.665	210.733	36.900	0.253
AOAC Fiber, g	23.475	8.032	22.608	7.425	0.595
Calcium, mg	1323.178	628.420	1153.995	583.843	0.104
Iron, mg	17.156	10.560	15.787	13.469	0.136
Magnesium, mg	382.583	105.165	392.803	135.416	0.951
Phosphorus, mg	1320.867	209.832	1272.319	274.088	0.206
Protassium, mg	3111.705	559.215	3152.619	518.152	0.659
Zinc, mg	23.457	22.583	16.931	19.173	**0.011**
Vitamin C, mg	301.606	352.025	250.314	325.117	0.281
Vitamin B1, mg	5.335	12.408	8.537	18.154	0.988
Vitamin B 2, mg	6.141	12.462	9.397	18.203	0.772
Niacin,	55.860	96.754	68.713	139.291	0.697
Vitamin B6, mg	17.589	47.516	17.995	35.439	0.978
Total Folate 1998 µg, with supplements	730.346	356.418	641.613	391.755	0.098
Saturated fat, g	20.914	5.048	21.560	5.644	0.612
Monosatuarated fat, g	23.940	8.099	28.095	10.674	**0.024**
Oleic, g	22.399	7.742	26.453	10.454	**0.023**
Polyunsaturated fat, g	14.544	3.336	15.714	4.419	0.224
Linoleic, g	12.254	2.936	13.238	3.798	0.227
Cholesterol, mg	239.243	109.288	234.799	103.888	0.896
Methionine, g	1.726	0.528	1.681	0.461	0.658
Vitamin D, IU	1413.131	1183.663	1301.196	1060.354	0.540
Alcohol, g	12.175	26.263	11.737	22.636	0.586
Caffeine, mg	133.516	130.469	150.350	120.124	0.183
Sacharin, mg	1.786	5.298	2.385	9.516	0.592
Vitamin B12, µg	146.312	304.390	96.950	243.680	0.181
Pantothenic acic, mg	15.054	14.876	16.666	20.230	0.558
Sucrose, g	37.708	11.603	40.326	13.978	0.355
Sodium, mg	2036.116	436.224	1833.650	402.204	**0.021**
Animal protein, g	49.185	19.337	48.418	18.046	0.791
Lactose, g	15.507	11.134	14.267	14.016	0.149
Tryptophan, g	0.832	0.196	0.807	0.178	0.487
Manganese, µg	5.107	1.884	4.416	2.261	**0.043**
Omega-3 (DHA + EPA) FA, g	0.319	0.323	0.388	0.321	0.430
Iodine, µm	63.382	66.877	34.258	75.004	**0.000**
Selenium, µm	16.470	22.873	10.472	24.074	**0.007**
Copper, mg	2.043	1.338	1.774	1.108	0.211
Fructose, g	21.041	8.600	21.214	8.480	0.875
Linolenic fatty acid, g	1.585	0.486	1.705	0.697	0.557
Butyric fatty acid, g	0.463	0.219	0.472	0.324	0.276
Caproic fatty acid, g	0.256	0.141	0.257	0.189	0.294

Caprylic fatty acid, g	0.197	0.105	0.196	0.120	0.523
Capric fatty acid, g	0.366	0.176	0.370	0.229	0.502
Lauric fatty acid, g	0.684	0.494	0.764	0.528	0.756
Myristic fatty acid, g	1.711	0.643	1.717	0.891	0.495
Pamitic fatty acid, g	11.444	2.524	11.822	2.502	0.433
Stearic fatty acid	4.823	1.271	5.026	1.360	0.484
Palmitoleic fatty acid, g	0.892	0.331	0.957	0.285	0.149
Eicosenoic fatty acid	0.242	0.084	0.299	0.141	0.061
Arachadonic fatty acid	0.180	0.100	0.220	0.123	0.210
Eicosapentaenoic fatty acid (EPA), g	0.124	0.156	0.133	0.137	0.881
Docosapentaenoic fatty acid (DPA)	0.030	0.022	0.043	0.036	0.274
Docosahexaenoic fatty acid (DHA)	0.196	0.170	0.248	0.192	0.337
Glutamate, g	14.583	2.834	13.931	2.815	0.213
Aspartate, g	7.022	1.596	6.948	1.473	0.836
Calcium, without supplements, µg	907.887	264.504	857.351	316.905	0.208
Iron, without supplements, µg	13.373	2.649	12.185	2.552	**0.028**
Zinc_without supplements, µg	11.040	2.130	10.090	1.917	**0.023**
Vitamin C, without supplements, µg	114.182	57.219	113.761	52.804	0.768
Thiamine Vitamin B1, without supplements, µg	1.510	0.606	1.308	0.216	**0.017**
Ribofalvin Vitamin B2, without supplements, µg	2.218	0.789	2.067	0.608	0.229
Pyridoxine Vitamin B6, without supplements, µg	2.126	0.416	1.997	0.379	0.082
Total Folat, without supplements, µg	689.244	630.021	655.752	614.667	0.121
Vitamin A, without supplements, IU	203.151	35.634	192.873	51.965	0.588
Vitamin B12, without supplements, µg	6.371	3.592	6.092	3.659	0.872
Alpha Carotene, µg	697.242	252.829	730.438	374.846	0.698
Beta Carotene, µg	7021.762	6227.619	7751.764	5704.023	0.823
Beta Cryptoxanthin, µg	230.193	207.615	233.219	144.279	0.628
Lycopene, µg	4621.280	3439.118	4578.212	4246.612	0.622
Lutein-Zeaxanthin, µg	3894.318	3124.539	4282.118	3391.117	0.595
Food Folat, 2001 µg	363.603	322.672	402.284	324.884	0.830
Folic Acid, 2001 µg	230.358	67.377	236.690	82.677	**0.024**
Long Chain N3 Fatty Acid 205+225+226	0.184	0.000	0.184	0.000	0.139
Retinol Equivalents of Vitamin A, µg	1649.495	1281.080	2541.855	2226.630	0.311
Total Vitamin E, mg, atoco includes supplements and fortified	23.342	25.899	19.475	14.617	0.222
Vitamin E atoco from food supplementation, only synthetic	0.096	0.077	0.079	0.064	0.952
Long Chain N3 Fatty Acid, witouht supplementation	2.153	5.101	1.605	3.702	0.158
Retinol Activity Equivalents, µg	1164.520	903.182	1750.315	1541.501	0.728
Total Vitamin E, atoco from Food Fortification only, Synthetic	11.110	4.740	11.697	5.412	0.035
Beta Carotene without supplementation,µg	4955.836	2700.301	6192.108	3469.777	0.627
Copper, without supplementation, mg	1.393	0.304	1.522	0.488	0.158
Phosphorus, without supplementation, mg	1254.203	274.030	1370.687	439.692	0.213
Potassium, without supplementation, mg	3164.499	506.929	3049.903	665.872	0.622
Magnesium, without supplmentation, mg	337.297	59.963	342.769	58.082	0.721
Manganese, without supplementation, mg	3.664	0.647	3.764	0.875	0.339
Maltose, g	1.483	0.000	1.483	0.000	0.677
Glucose, g	21.391	11.311	24.965	19.413	0.857
Total sugars, g	98.847	36.091	100.257	38.268	0.756
c9, t11 conjug diene isomer 18:2 Linoleic, mg	78.428	28.161	73.771	38.370	0.130
Total Trans, 2001	0.933	0.246	0.896	0.364	0.240
Threonine, g	2.909	0.772	2.847	0.693	0.677

Isoleucine, g	3.438	0.937	3.327	0.811	0.525
Leucine, g	5.930	1.457	5.746	1.354	0.483
Lysine, g	5.262	1.527	5.155	1.439	0.695
Cystine, g	0.956	0.223	0.902	0.175	0.199
Phenylalanine, g	3.410	0.793	3.292	0.700	0.430
Tyrosine, g	2.681	0.702	2.598	0.661	0.493
Valine, g	4.009	1.033	3.881	0.928	0.492
Arginine, g	4.284	1.019	4.247	0.918	0.897
Histindine, g	2.110	0.517	2.048	0.463	0.530
Alanine, g	3.610	0.989	3.523	0.862	0.657
Glycine, g	3.175	0.822	3.072	0.716	0.511
Proline, g	4.900	1.038	4.574	1.106	0.082
Serine, g	3.635	0.866	3.519	0.790	0.476
Glycemic Load	115.896	27.108	107.392	24.170	0.135
Glycemic Index	52.116	4.273	50.641	4.899	0.103
Bran Score, g	10.183	10.495	7.091	6.178	**0.011**
Added Bran from wheat, rice, oat, corn, g	3.439	7.130	1.953	2.279	**0.021**
Natural Bran, G	6.768	4.440	5.164	4.218	**0.010**
Whole grain amount, g	42.239	27.532	32.622	21.837	**0.031**
Whole Grain Score without added Bran and Germ, g	38.708	22.665	30.669	20.041	**0.036**
Dairy Protein, g	16.276	7.024	15.736	10.905	0.132
Diary Fat, g	13.492	6.419	13.452	9.486	0.207
Dariy Calcium, mg	504.174	235.157	476.544	341.482	0.099
Diary Vitamin D, IU	117.678	104.803	106.162	108.244	0.146
Free Choline, choline-contributing metabolite, mg	75.308	17.723	77.376	21.408	0.698
Choline from Glycerophosphocholine, mg	56.933	17.358	57.305	26.165	0.577
Choline from Phosphocholine, mg	14.781	5.065	15.517	5.595	0.585
Choline from Phosphatidylcholine, mg	179.256	75.332	183.346	76.918	0.655
Choline from Sphingomyeline, mg	17.692	7.296	17.068	7.042	0.576
Total Cholline, mg, no betaine	343.537	89.622	349.777	101.939	0.806
Free Choline, without supplementation, mg	73.924	16.293	72.665	16.656	0.661
Choline from Phosphatidylcholine, without supplments, mg	179.080	74.970	180.149	71.111	0.744
Choline, without supplements, mg	341.951	88.423	341.862	92.539	0.975
Total Omega 3, g, Sacks, 2001	1.880	0.672	2.096	0.858	0.288
Omega 6, mg, Sacks, 2001	13.298	3.006	14.323	3.833	0.206
Long Cahin Fatty Acid, g, Sacks, 2001	0.355	0.338	0.443	0.352	0.320
Omega 6, no gamma, g, Sacks, 2001	13.224	3.002	14.253	3.842	0.206
Alpha Linolenic fatty acid, g, Sacks, 2001	1.528	0.506	1.666	0.713	0.472
Gamma Linolenic fatty acid, g, Sacks 2001	0.016	0.009	0.017	0.007	0.296
Alpha+Gamma Linolenic, g, Sacks, 2001	1.544	0.505	1.681	0.715	0.484
Total Omega 3, without supplments, g, Sacks 2001	1.687	0.494	1.925	0.719	0.121
Omega 6, without supplements,g, Sacks 2011	13.279	3.008	14.304	3.826	0.204
Long Chain Fatty Acid, without supplementation, g	0.228	0.172	0.345	0.300	0.119
n611_wo	13.205	3.004	14.234	3.834	0.204
Phylloquinone Vitamin K1, µg	196.018	161.844	215.065	144.303	0.478
Phenylalanine, from Aspartame, g	0.023	0.054	0.022	0.062	0.859
Aspartic Acid, from Aspartame, g	0.023	0.054	0.022	0.062	0.859
Aspartame, 2006	0.046	0.107	0.043	0.123	0.944
Acrylamide, µg	19.807	9.319	17.085	7.321	0.146
Betaine, Choline derivates, mg	109.754	42.954	98.677	46.656	0.117
Sum of Betaine and Choline, mg	453.274	86.874	448.258	106.357	0.650
Quercetin, mg, flavonol USDA 2007	15.441	8.436	16.112	8.586	0.439
Kaempferol, mg, flavonol USDA 2007	4.092	3.474	3.770	3.051	0.841
Myricetin, mg, Flavonol USDA, 2007	1.935	1.281	1.868	1.103	0.868
Isorhamnetin, mg, flavonol USDA, 2007	1.311	1.331	1.441	1.276	0.118
Total USDA Flavonols, 2007	22.765	12.076	23.123	12.115	0.660

Apigenin, mg, Flavone USDA, 2007	1.072	1.458	0.949	1.383	0.962
Luteolin, mg, Flavone USDA, 207	0.820	0.462	0.918	0.629	0.358
Total USDA Flavones, 2007	1.893	1.631	1.856	1.491	0.796
Hesperetin, mg flavanone USDA, 2007	19.197	18.944	20.653	31.281	0.794
Naringenin, mg flavone USDA, 2007	8.860	10.626	9.492	10.708	0.506
Eriodictyol, mgflavone, USDA, 2007	0.067	0.117	0.084	0.131	0.196
Total USDA Flavanone, 2008	28.264	27.404	29.043	36.456	0.650
Catechin, mg, flavan-3-ol USDA, 2007	17.736	11.387	16.132	11.593	0.378
Callocatechin, mg flavon-3-ol USDA, 2007	1.717	2.784	1.739	3.097	0.805
Epicatechin, mg, flavan-3-ol USDA, 2007	11.988	7.087	13.454	8.869	0.364
Epigallocatechin mg, flavan-3-ol USDA 2007	10.769	17.320	10.622	17.435	0.520
Epicatechin 3-gallate mg, flavan-3-ol USDA 2007	7.711	13.371	7.896	14.578	0.664
Epigallocatechin 3-gallate mg flavan-3-ol USDA 2007	12.306	21.032	12.640	22.906	0.443
TOTAL USDA FLAVAN-3-OLS, 2007	61.690	61.681	60.825	64.455	0.960
Cyanidin mg, anthocyanidin USDA 2007	7.789	10.915	7.846	8.310	0.760
Delphinidin mg, anthocyanidin USDA 2007	8.125	8.387	7.984	9.171	0.489
Malvidin mg, anthocyanidin USDA 2007	12.796	12.318	12.302	13.542	0.455
Pelargonidin mg, anthocyanidin USDA 2007	5.649	7.160	5.205	5.570	0.476
Peonidin mg, anthocyanidin USDA 2007	4.595	4.733	4.463	5.175	0.427
TOTAL USDA ANTHOCYANIDINS, 2007	41.021	37.496	39.027	36.582	0.656
Proanthocyanidin, monomers, USDA, 2007	28.974	24.467	31.842	26.349	0.319
Theaflavin mg, flavan-3-ol USDA 2007	2.003	3.473	1.969	3.603	0.928
Theaflavin 3-gallate mg, flavan-3-ol USDA 2007	1.582	2.740	1.546	2.822	0.932
Theaflavin 3'-gallate mg, flavan-3-ol USDA 2007	1.917	3.320	1.882	3.442	0.930
Theaflavin 3,3' digallate mg, flavan-3-ol USDA 2007	2.224	3.857	2.193	4.019	0.930
Thearubigins mg, flavan-3-ol USDA 2007	136.933	266.952	169.363	344.519	0.668
Proanthocyanidin, dimers, USDA, 2007	27.566	17.378	30.363	19.147	0.332
Proanthocyanidin, trimers, USDA, 2007	13.995	6.408	16.238	8.215	0.143
Proanthocyanidin, 4-6mers, USDA, 2007	36.130	19.442	43.823	26.456	0.121
Proanthocyanidin, 7-10mers, USDA, 2007	24.093	15.076	29.366	20.837	0.159
Proanthocyanidin, polymers, USDA, 2007	66.086	43.758	74.317	52.947	0.564
Total Theaflavin and Polymers Proanthocyanidins, USDA, 2007	281.990	237.107	308.724	256.325	0.375
USDA FLAVONOIDS NO PROANTHOCYANIDINS	266.374	264.510	257.966	255.044	0.829
Betaine without supplements, choline derivative mg	109.750	42.955	98.671	46.657	0.116
Phylloquinone Vitamin K1 without supplements, mcg	181.079	153.449	208.177	147.175	0.337
Quercetin without supplements mg, flavonol USDA 2007	15.441	8.436	16.112	8.586	0.439
Vegetable protein, g	27.161	6.705	25.257	7.313	0.112
Added Sugar, gm	42.689	20.079	43.664	23.633	0.993
Natural Sugar, g	52.760	18.815	54.831	20.190	0.738
Sucralose sweetener g	1.308	3.669	0.804	2.718	0.984
Beta Tocopherol mg, USDA 2008	0.291	0.098	0.262	0.071	0.166
Gamma Tocopherol mg, USDA 2008	8.162	2.508	8.598	2.778	0.479
Delta Tocopherol mg, USDA 2008	1.747	0.720	1.729	0.777	0.791
Alpha Tocotrienol mg, USDA 2008	1.017	0.571	0.877	0.319	0.188

Beta Tocotrienol mg, USDA 2008	0.996	0.719	0.678	0.391	0.014
Gamma Tocotrienol mg, USDA 2008	0.489	0.256	0.499	0.187	0.455
Delta Tocotrienol mg, USDA 2008	0.055	0.049	0.043	0.022	0.291
mg Total Tocopherols without supplement 2008	23.282	6.982	25.940	9.129	0.142
Sum of Betaine and Choline, without supplements mg	451.528	85.556	440.093	98.659	0.441
Alpha Carotene, without supplements µg	872.452	773.780	963.154	708.723	0.698
Beta Cryptoxanthin, without supplements µg	245.982	183.058	243.689	226.039	0.628
Lycopene, without supplements µg	4573.084	3713.023	5098.315	4040.560	0.524
Lutein and Zeaxanthin, without supplements µg	3346.473	3030.112	3906.159	3182.273	0.439
Omega 3 (f205+f226),no alpha-linolenic acid, without supplements mg	0.192	0.151	0.291	0.260	0.181
Discretionary Sold Fat, gm/100 grams, 2009	20.588	6.906	20.336	9.861	0.379
Discretionary Liquid Fat, gms/100 grams, 2009	26.626	9.851	32.413	16.054	0.056
Insulinogenic Load, Glucose	711.710	119.788	667.792	135.074	0.077
Dietary Insulin Index , Glucose, (il*100)/calor	39.539	6.655	37.100	7.504	0.077
AOAC fiber gms, 1993, from fruit	5.246	3.488	6.131	3.912	0.442
AOAC fiber g, 1993, from vegetable	6.469	3.778	7.013	3.920	0.558
AOAC fiber gms, 1993, from cruciferous	0.984	1.095	1.018	1.002	0.972
AOAC fiber gms, 1993, from legume	2.173	1.297	1.978	1.455	0.234
AOAC fiber gms, 1993, from cereal	7.590	5.777	5.390	2.838	**0.008**
Daidzein, Phytoestrogen, Isoflavone, mg	1.201	1.809	1.049	1.455	0.881
Genistein, Phytoestrogen, Isoflavone, mg	1.635	3.130	1.255	1.765	0.897
Glycitein, Phytoestrogen, Isoflavone, mg	0.318	0.563	0.307	0.628	0.831
Formononetin, Phytoestrogen mg, Isoflavones	0.037	0.051	0.033	0.029	0.869
Biochanin A, Phytoestrogen mg, Isoflavones	0.038	0.031	0.043	0.044	0.540
Total Isoflavones, No Bioch or Formo, mg	3.162	5.473	2.617	3.842	0.984
Total Isoflavones, Including Bioch and Formo, mg	3.242	5.496	2.687	3.839	0.990
