# Supplementary material for: Fuchs Endothelial Corneal Dystrophy Associations with Systemic Disease, Lifestyle, and Nutritional Intake
Source: Ophthalmol Sci. 2025 Jul 31;6(1):100899. doi: 10.1016/j.xops.2025.100899 (PMC12478083; doi:10.1016/j.xops.2025.100899)
Supplement: Appendix 2 [file mmc2.pdf]

Supplemental Appendix 2: Lifestyle Questionnaire for Exercise and Smoking Behavior

Massachusetts Eye and Ear Infirmary

Fuchs Endothelial Corneal Dystrophy Nutrition Study

Smoking/Exercise Assessment Questionnaire

1. **On average, how many hours do you exercise per week?**
   _______ Hours/week

2. **Have you ever smoked tobacco (cigarettes, pipes, cigars)?**

   □ No (please return survey)
   □ Yes

3. **At what age did you start smoking?**
   _______ years old

4. **Do you currently smoke?**

   □ No
   □ Yes

5. **About how many years were you (or have you been) a smoker?**
   ________ years

6. **On average, how many cigarettes did you (or do you currently) smoke per day?**
   ________ cigarettes/day
