# Supplementary material for: Fuchs Endothelial Corneal Dystrophy Associations with Systemic Disease, Lifestyle, and Nutritional Intake
Source: Ophthalmol Sci. 2025 Jul 31;6(1):100899. doi: 10.1016/j.xops.2025.100899 (PMC12478083; doi:10.1016/j.xops.2025.100899)
Supplement: Appendix 1 [file mmc3.pdf]

DIETARY ASSESSMENT

ID NUMBER: ____________________

USE NO. 2 PENCIL ONLY 2

- Darken one circle per question that corresponds to your answer
- Follow arrows

VITAMINS

1. Do you currently take **multi-vitamins**? (Please report other individual vitamins in question 2.)

◯ No
◯ Yes

a) How many do you take per week?

◯ 2 or less ◯ 3–5 ◯ 6–9 ◯ 10 or more

b) For how many years have you been taking them?

◯ 1 year or less ◯ 2–4 years ◯ 5–9 years ◯ 10 or more years

c) What specific brand (or equivalency) do you usually take?

◯ Centrum Silver ◯ Centrum ◯ Other
◯ Theragran M ◯ One-A-Day Essential

e.g., AARP Alphabet II Formula 643 Multivitamins and Minerals

2. Not counting multi-vitamins, do you currently take any of the following specific vitamins or minerals? DO NOT report content of multi-vitamins mentioned above!

Vitamin A

◯ **No**
◯ Yes, **seasonal only**
◯ Yes, **currently** take it most months

Dose per day?

◯ Less than 10,000 IU
◯ 10,000 to 15,000 IU
◯ 16,000 to 22,000 IU
◯ 23,000 IU or more
◯ Don't know

How long?

◯ 0–1 year
◯ 2–4 years
◯ 5–9 years
◯ 10 years or more

Beta Carotene

◯ **No**
◯ Yes, **seasonal only**
◯ Yes, **currently** take it most months

Dose per day?

◯ Less than 10,000 IU
◯ 10,000 to 15,000 IU
◯ 16,000 to 22,000 IU
◯ 23,000 IU or more
◯ Don't know

How long?

◯ 0–1 year
◯ 2–4 years
◯ 5–9 years
◯ 10 years or more

PLEASE DO NOT WRITE IN THIS AREA

SERIAL #

PROOF

3/8" spine perf

2. (Continued) **Not counting multi-vitamins,** do you currently take any of the following specific vitamins or minerals? DO NOT report content of multi-vitamin question.

Vitamin B_6
- ◯ No
- ◯ Yes, **currently** take it

Dose per day?
- ◯ Less than 50 mg
- ◯ 50 to 99 mg
- ◯ 100 to 149 mg
- ◯ 150 mg or more
- ◯ Don't know

How long?
- ◯ 0–1 year
- ◯ 2–4 years
- ◯ 5–9 years
- ◯ 10 years or more

B6 d l

Vitamin C
- ◯ No
- ◯ Yes, **seasonal only**
- ◯ Yes, **currently** take it most months

Dose per day?
- ◯ Less than 400 mg
- ◯ 400 to 700 mg
- ◯ 750 to 1,250 mg
- ◯ 1,300 mg or more
- ◯ Don't know

How long?
- ◯ 0–1 year
- ◯ 2–4 years
- ◯ 5–9 years
- ◯ 10 years or more

C d l

Vitamin D
(In calcium supplements or separately)
- ◯ No
- ◯ Yes, **seasonal only**
- ◯ Yes, **currently** take it most months

Dose per day?
- ◯ Less than 300 IU
- ◯ 300 to 500 IU
- ◯ 600 to 900 IU
- ◯ 1,000 IU or more
- ◯ Don't know

How long?
- ◯ 0–1 year
- ◯ 2–4 years
- ◯ 5–9 years
- ◯ 10 years or more

D d l

Vitamin E
- ◯ No
- ◯ Yes, **currently** take it

Dose per day?
- ◯ Less than 100 IU
- ◯ 100 to 250 IU
- ◯ 300 to 500 IU
- ◯ 600 IU or more
- ◯ Don't know

How long?
- ◯ 0–1 year
- ◯ 2–4 years
- ◯ 5–9 years
- ◯ 10 years or more

Type:
- ◯ Natural
- ◯ Regular (dl)
- ◯ Unknown

E d l

Selenium
- ◯ No
- ◯ Yes, **currently** take it

Dose per day?
- ◯ Less than 80 mcg
- ◯ 80 to 130 mcg
- ◯ 140 to 250 mcg
- ◯ 260 mcg or more
- ◯ Don't know

How long?
- ◯ 0–1 year
- ◯ 2–4 years
- ◯ 5–9 years
- ◯ 10 years or more

S d l

Iron
- ◯ No
- ◯ Yes, **currently** take it

Dose per day?

mg of elemental iron (325 mg Ferrous Sulfate = 65 mg elemental iron)
- ◯ Less than 20 mg
- ◯ 20 to 39 mg
- ◯ 40 to 49 mg
- ◯ 50 mg or more
- ◯ Don't know

How long?
- ◯ 0–1 year
- ◯ 2–4 years
- ◯ 5–9 years
- ◯ 10 years or more

I d l

Zinc
- ◯ No
- ◯ Yes, **currently** take it

Dose per day?
- ◯ Less than 25 mg
- ◯ 25 to 74 mg
- ◯ 75 to 100 mg
- ◯ 101 mg or more
- ◯ Don't know

How long?
- ◯ 0–1 year
- ◯ 2–4 years
- ◯ 5–9 years
- ◯ 10 years or more

Z d l

PROOF

3/8" spine perf

2. (Continued) **Not counting multi-vitamins,** do you currently take any of the following specific vitamins or minerals? DO NOT report content of multi-vitamin question.

Calcium or Dolomite (Include Tums)

- ◯ **No**
- ◯ Yes, **currently** take it

Dose per day? (Include elemental Calcium in Tums)

- ◯ Less than 600 mg
- ◯ 600 to 900 mg
- ◯ 901 to 1,500 mg
- ◯ 1,501 mg or more
- ◯ Don't know

How long?

- ◯ 0–1 year
- ◯ 2–4 years
- ◯ 5–9 years
- ◯ 10 years or more

Fish Oil (Omega 3 fatty acids)

- ◯ **No**
- ◯ Yes, **currently** take it

Dose per day?

- ◯ Less than 800 mg
- ◯ 800 to 1499 mg
- ◯ 1500 to 2499 mg
- ◯ 2500 mg or more
- ◯ Don't know

How long?

- ◯ 0–1 year
- ◯ 2–4 years
- ◯ 5–9 years
- ◯ 10 years or more

Potassium

- ◯ **No**
- ◯ Yes, **currently** take it

Dose per day?

- ◯ Less than 2.5 mEq (100 mg)
- ◯ 3 to 10 mEq
- ◯ 11 to 20 mEq
- ◯ 21 mEq or more
- ◯ Don't know

How long?

- ◯ 0–1 year
- ◯ 2–4 years
- ◯ 5–9 years
- ◯ 10 years or more

PROOF

Which other supplements are you taking currently on a regular basis (at least once per week)?

- ◯ None
- ◯ Metamucil
- ◯ Cod liver oil
- ◯ Brewer's yeast
- ◯ Folic acid or folate (B_9)
- ◯ Magnesium
- ◯ Niacin
- ◯ Vitamin B_{12}
- ◯ Flaxseed oil
- ◯ Flaxseed
- ◯ B-complex
- ◯ Melatonin
- ◯ Chromium
- ◯ Lecithin
- ◯ CoEnzyme Q_{10}
- ◯ Choline
- ◯ Evening primrose
- ◯ Ginkgo biloba
- ◯ Lycopene
- ◯ DHEA
- ◯ Glucosamine/Chondroitin
- ◯ Other Supplements (specify) ____________

DAIRY FOODS

In the following section, please describe how often on average you have used the amount specified in the past year. Please indicate your average total use, taking the portion size into account. For example, if you use 1/2 a glass of milk twice a week, mark 1 glass per week to represent your average total intake.

3. For each food listed, fill in the circle indicating your average total use of the amount specified during the past year.

Skim milk (8 oz. glass)	1% or 2% milk (8 oz. glass)	Whole milk (8 oz. glass)
◯ Never	◯ Never	◯ Never
◯ Less than once per month	◯ Less than once per month	◯ Less than once per month
◯ 1–3 glasses per month	◯ 1–3 glasses per month	◯ 1–3 glasses per month
◯ 1 glass per week	◯ 1 glass per week	◯ 1 glass per week
◯ 2–4 glasses per week	◯ 2–4 glasses per week	◯ 2–4 glasses per week
◯ 5–6 glasses per week	◯ 5–6 glasses per week	◯ 5–6 glasses per week
◯ 1 glass per day	◯ 1 glass per day	◯ 1 glass per day
◯ 2–3 glasses per day	◯ 2–3 glasses per day	◯ 2–3 glasses per day
◯ 4 or more glasses per day	◯ 4 or more glasses per day	◯ 4 or more glasses per day

3/8" spine perf

3. (Continued) Please fill in your average total use, during the past year, of each specified food.

Soy milk (8 oz. glass)
- ◯ Never
- ◯ Less than once per month
- ◯ 1–3 glasses per month
- ◯ 1 glass per week
- ◯ 2–4 glasses per week
- ◯ 5–6 glasses per week
- ◯ 1 glass per day
- ◯ 2–3 glasses per day
- ◯ 4 or more glasses per day

Cream, e.g., in coffee, whipped or sour cream (1 tbs.)
- ◯ Never
- ◯ Less than once per month
- ◯ 1–3 tbs. per month
- ◯ 1 tbs. per week
- ◯ 2–4 tbs. per week
- ◯ 5–6 tbs. per week
- ◯ 1 tbs. per day
- ◯ 2 or more tbs. per day

Non-dairy coffee whitener (1 tbs.)
- ◯ Never
- ◯ Less than once per month
- ◯ 1–3 tbs. per month
- ◯ 1 tbs. per week
- ◯ 2–4 tbs. per week
- ◯ 5–6 tbs. per week
- ◯ 1 tbs. per day
- ◯ 2 or more tbs. per day

Frozen yogurt, sherbet or low-fat ice cream (1 cup)
- ◯ Never
- ◯ Less than once per month
- ◯ 1–3 times per month
- ◯ Once per week
- ◯ 2–4 times per week
- ◯ 5–6 times per week
- ◯ Once per day
- ◯ 2 or more servings per day

Regular ice cream (1 cup)
- ◯ Never
- ◯ Less than once per month
- ◯ 1–3 times per month
- ◯ Once per week
- ◯ 2–4 times per week
- ◯ 5–6 times per week
- ◯ Once per day
- ◯ 2 or more servings per day

Flavored yogurt, sweetened with fruit or other flavoring (1 cup)
- ◯ Never
- ◯ Less than once per month
- ◯ 1–3 cups per month
- ◯ 1 cup per week
- ◯ 2–4 cups per week
- ◯ 5–6 cups per week
- ◯ 1 cup per day
- ◯ 2 or more servings per day

PROOF

Yogurt, low carb, artificially sweetened or plain (1 cup)
- ◯ Never
- ◯ Less than once per month
- ◯ 1–3 cups per month
- ◯ 1 cup per week
- ◯ 2–4 cups per week
- ◯ 5–6 cups per week
- ◯ 1 cup per day
- ◯ 2 or more servings per day

What type of yogurt do you usually eat?
- ◯ None
- ◯ Regular
- ◯ Low fat
- ◯ Nonfat

Cottage or ricotta cheese (1/2 cup)
- ◯ Never
- ◯ Less than once per month
- ◯ 1–3 times per month
- ◯ Once per week
- ◯ 2–4 times per week
- ◯ 5–6 times per week
- ◯ Once per day
- ◯ 2 or more servings per day

Cream cheese (1 oz.)
- ◯ Never
- ◯ Less than once per month
- ◯ 1–3 times per month
- ◯ Once per week
- ◯ 2–4 times per week
- ◯ 5–6 times per week
- ◯ Once per day
- ◯ 2 or more servings per day

Other cheese, e.g., American, cheddar, etc., plain or as part of a dish (1 slice or 1 oz. serving)
- ◯ Never
- ◯ Less than once per month
- ◯ 1–3 slices per month
- ◯ 1 slice per week
- ◯ 2–4 slices per week
- ◯ 5–6 slices per week
- ◯ 1 slice per day
- ◯ 2 or more slices per day

What type of cheese do you usually eat?
- ◯ None
- ◯ Regular
- ◯ Low fat or lite
- ◯ Nonfat

3/8" spine perf

3. (Continued) Please fill in your average total use, during the past year, of each specified food.

Pure butter (small pat or tsp.), added to food or bread; exclude use in cooking

- ◯ Never
- ◯ Less than once per month
- ◯ 1–3 pats per month
- ◯ 1 pat per week
- ◯ 2–4 pats per week
- ◯ 5–6 pats per week
- ◯ 1 pat per day
- ◯ 2–3 pats per day
- ◯ 4 or more pats per day

"Spreadable butter"—butter/oil blend (small pat or tsp.), added to food or bread; exclude use in cooking

- ◯ Never
- ◯ Less than once per month
- ◯ 1–3 pats per month
- ◯ 1 pat per week
- ◯ 2–4 pats per week
- ◯ 5–6 pats per week
- ◯ 1 pat per day
- ◯ 2–3 pats per day
- ◯ 4 or more pats per day

Margarine or spread (small pat or tsp.), added to food or bread; exclude use in cooking

- ◯ Never
- ◯ Less than once per month
- ◯ 1–3 pats per month
- ◯ 1 pat per week
- ◯ 2–4 pats per week
- ◯ 5–6 pats per week
- ◯ 1 pat per day
- ◯ 2–3 pats per day
- ◯ 4 or more pats per day

What form of margarine or spreadable butter do you usually use? (Exclude pure butter)

◯ None

Form? ◯ Stick ◯ Tub ◯ Spray ◯ Squeeze (liquid)

Type? ◯ Regular ◯ Light spread ◯ Nonfat

What specific **brand** and **type** (e.g., Shedd's Spread Country Crock Light Tub)?

0 1 2 3 4 5 6 7 8 9 | 0 1 2 3 4 5 6 7 8 9 | 0 1 2 3 4 5 6 7 8 9

f t b

PROOF

3/8" spine perf

FRUITS

4. Please fill in your average total use, during the past year, of each specified food.

Please try to average your seasonal use of foods over the entire year. For example, if a food such as cantaloupe is eaten 4 times a week during the 3 months that it is in season, then the average total use would be once per week over the year.

Raisins (1 oz. or small pack) or grapes (1/2 cup)

- ◯ Never
- ◯ Less than once per month
- ◯ 1–3 times per month
- ◯ Once per week
- ◯ 2–4 times per week
- ◯ 5–6 times per week
- ◯ Once per day
- ◯ 2 or more servings per day

Prunes or dried plums (6 prunes or 1/4 cup)

- ◯ Never
- ◯ Less than once per month
- ◯ 1–3 times per month
- ◯ Once per week
- ◯ 2–4 times per week
- ◯ 5–6 times per week
- ◯ Once per day

Prune Juice (small glass)

- ◯ Never
- ◯ Less than once per month
- ◯ 1–3 glasses per month
- ◯ 1 glass per week
- ◯ 2–4 glasses per week
- ◯ 5–6 glasses per week
- ◯ 1 glass per day
- ◯ 2 or more glasses per day

Bananas (1)

- ◯ Never
- ◯ Less than once per month
- ◯ 1–3 per month
- ◯ 1 per week
- ◯ 2–4 per week
- ◯ 5–6 per week
- ◯ 1 per day
- ◯ 2 or more per day

Cantaloupe (1/4 melon)

- ◯ Never
- ◯ Less than once per month
- ◯ 1–3 times per month
- ◯ Once per week
- ◯ 2–4 times per week
- ◯ 5–6 times per week
- ◯ Once per day
- ◯ 2–3 times per day
- ◯ 4 or more servings per day

Avocado (1/2 fruit or 1/2 cup)

- ◯ Never
- ◯ Less than once per month
- ◯ 1–3 times per month
- ◯ Once per week
- ◯ 2–4 times per week
- ◯ 5–6 times per week
- ◯ One per day
- ◯ Two or more per day

4

4. (Continued) Please fill in your average total use, during the past year, of each specified food.

Applesauce (1/2 cup)
- ◯ Never
- ◯ Less than once per month
- ◯ 1–3 times per month
- ◯ Once per week
- ◯ 2–4 times per week
- ◯ 5–6 times per week
- ◯ One or more per day

Fresh apples or pears (1)
- ◯ Never
- ◯ Less than once per month
- ◯ 1–3 per month
- ◯ 1 per week
- ◯ 2–4 per week
- ◯ 5–6 per week
- ◯ 1 per day
- ◯ 2–3 per day
- ◯ 4 or more per day

Apple juice or cider (small glass)
- ◯ Never
- ◯ Less than once per month
- ◯ 1–3 glasses per month
- ◯ 1 glass per week
- ◯ 2–4 glasses per week
- ◯ 5–6 glasses per week
- ◯ 1 glass per day
- ◯ 2 or more glasses per day

Oranges (1)
- ◯ Never
- ◯ Less than once per month
- ◯ 1–3 per month
- ◯ 1 per week
- ◯ 2–4 per week
- ◯ 5–6 per week
- ◯ 1 per day
- ◯ 2–3 per day
- ◯ 4 or more per day

Orange juice—calcium fortified (small glass)
- ◯ Never
- ◯ Less than once per month
- ◯ 1–3 glasses per month
- ◯ 1 glass per week
- ◯ 2–4 glasses per week
- ◯ 5–6 glasses per week
- ◯ 1 glass per day
- ◯ 2 or more glasses per day

Orange juice—regular (not calcium fortified) (small glass)
- ◯ Never
- ◯ Less than once per month
- ◯ 1–3 glasses per month
- ◯ 1 glass per week
- ◯ 2–4 glasses per week
- ◯ 5–6 glasses per week
- ◯ 1 glass per day
- ◯ 2 or more glasses per day

Grapefruit (1/2)
- ◯ Never
- ◯ Less than once per month
- ◯ 1–3 times per month
- ◯ Once per week
- ◯ 2–4 times per week
- ◯ 5–6 times per week
- ◯ Once per day
- ◯ 2–3 times per day
- ◯ 4 or more times per day

Grapefruit juice (small glass)
- ◯ Never
- ◯ Less than once per month
- ◯ 1–3 glasses per month
- ◯ 1 glass per week
- ◯ 2–4 glasses per week
- ◯ 5–6 glasses per week
- ◯ 1 glass per day
- ◯ 2 or more glasses per day

Other fruit juices (small glass)
- ◯ Never
- ◯ Less than once per month
- ◯ 1–3 glasses per month
- ◯ 1 glass per week
- ◯ 2–4 glasses per week
- ◯ 5–6 glasses per week
- ◯ 1 glass per day
- ◯ 2 or more glasses per day

Strawberries, fresh, frozen or canned (1/2 cup)
- ◯ Never
- ◯ Less than once per month
- ◯ 1–3 times per month
- ◯ Once per week
- ◯ 2–4 times per week
- ◯ 5–6 times per week
- ◯ Once or more per day

Blueberries, fresh, frozen or canned (1/2 cup)
- ◯ Never
- ◯ Less than once per month
- ◯ 1–3 times per month
- ◯ Once per week
- ◯ 2–4 times per week
- ◯ 5 or more servings per week

Peaches or plums (1 fresh or 1/2 cup canned)
- ◯ Never
- ◯ Less than once per month
- ◯ 1–3 per month
- ◯ Once per week
- ◯ 2–4 per week
- ◯ 5–6 per week
- ◯ 1 or more per day

Apricots (1 fresh, 1/2 cup canned or 5 dried)
- ◯ Never
- ◯ Less than once per month
- ◯ 1–3 per month
- ◯ Once per week
- ◯ 2–4 per week
- ◯ 5 or more servings per week

PROOF

3/8" spine perf

VEGETABLES

5. Please fill in your average total use, during the past year, of each specified food.

Tomatoes (2 slices)
- ◯ Never
- ◯ Less than once per month
- ◯ 1–3 per month
- ◯ 1 per week
- ◯ 2–4 per week
- ◯ 5–6 per week
- ◯ 1 or more per day

Tomato or V8 juice (small glass)
- ◯ Never
- ◯ Less than once per month
- ◯ 1–3 glasses per month
- ◯ 1 glass per week
- ◯ 2–4 glasses per week
- ◯ 5–6 glasses per week
- ◯ 1 glass per day
- ◯ 2 or more glasses per day

Tomato sauce (1/2 cup) e.g., spaghetti sauce
- ◯ Never
- ◯ Less than once per month
- ◯ 1–3 times per month
- ◯ Once per week
- ◯ 2–4 times per week
- ◯ 5 or more servings per week

Salsa, picante or taco sauce (1/4 cup)
- ◯ Never
- ◯ Less than once per month
- ◯ 1–3 times per month
- ◯ Once per week
- ◯ 2–4 times per week
- ◯ 5–6 times per week
- ◯ Once per day
- ◯ 2 or more servings per day

Tofu, soy burgers, soybeans, miso, or other soy protein (3–4 oz.)
- ◯ Never
- ◯ Less than once per month
- ◯ 1–3 times per month
- ◯ Once per week
- ◯ 2–4 times per week
- ◯ 5–6 times per week
- ◯ Once per day
- ◯ 2 or more servings per day

String beans (1/2 cup)
- ◯ Never
- ◯ Less than once per month
- ◯ 1–3 times per month
- ◯ Once per week
- ◯ 2–4 times per week
- ◯ 5 or more servings per week

Broccoli (1/2 cup)
- ◯ Never
- ◯ Less than once per month
- ◯ 1–3 times per month
- ◯ Once per week
- ◯ 2–4 times per week
- ◯ 5–6 times per week
- ◯ 1 or more servings per day

Cabbage or cole slaw (1/2 cup)
- ◯ Never
- ◯ Less than once per month
- ◯ 1–3 times per month
- ◯ Once per week
- ◯ 2–4 times per week
- ◯ 5–6 times per week
- ◯ 1 or more servings per day

Cauliflower (1/2 cup)
- ◯ Never
- ◯ Less than once per month
- ◯ 1–3 times per month
- ◯ Once per week
- ◯ 2–4 times per week
- ◯ 5–6 times per week
- ◯ 1 or more servings per day

Brussels sprouts (1/2 cup)
- ◯ Never
- ◯ Less than once per month
- ◯ 1–3 times per month
- ◯ Once per week
- ◯ 2–4 times per week
- ◯ 5–6 times per week
- ◯ 1 or more servings per day

Carrots, raw (1/2 carrot or 2–4 sticks)
- ◯ Never
- ◯ Less than once per month
- ◯ 1–3 times per month
- ◯ Once per week
- ◯ 2–4 times per week
- ◯ 5–6 times per week
- ◯ Once per day
- ◯ 2 or more servings per day

Carrots, cooked (1/2 cup) or carrot juice (2–3 oz.)
- ◯ Never
- ◯ Less than once per month
- ◯ 1–3 times per month
- ◯ Once per week
- ◯ 2–4 times per week
- ◯ 5–6 times per week
- ◯ Once per day
- ◯ 2 or more servings per day

Corn (1 ear or 1/2 cup frozen or canned)
- ◯ Never
- ◯ Less than once per month
- ◯ 1–3 per month
- ◯ 1 per week
- ◯ 2–4 per week
- ◯ 5–6 per week
- ◯ 1 or more servings per day

Peas or lima beans (1/2 cup fresh, frozen or canned)
- ◯ Never
- ◯ Less than once per month
- ◯ 1–3 times per month
- ◯ Once per week
- ◯ 2–4 times per week
- ◯ 5–6 times per week
- ◯ 1 or more servings per day

Mixed vegetables, stir fry (1/2 cup), vegetable soup (1 cup)
- ◯ Never
- ◯ Less than once per month
- ◯ 1–3 times per month
- ◯ Once per week
- ◯ 2–4 times per week
- ◯ 5–6 times per week
- ◯ 1 or more servings per day

PROOF

3/8" spine perf

5

5. (Continued) Please fill in your average total use, during the past year, of each specified food.

Beans or lentils, baked, dried or soup (1/2 cup)
- ◯ Never
- ◯ Less than once per month
- ◯ 1–3 times per month
- ◯ Once per week
- ◯ 2–4 times per week
- ◯ 5–6 times per week
- ◯ 1 or more servings per day

Dark orange (winter) squash (1/2 cup)
- ◯ Never
- ◯ Less than once per month
- ◯ 1–3 times per month
- ◯ Once per week
- ◯ 2–4 times per week
- ◯ 5–6 times per week
- ◯ 1 or more servings per day

Eggplant, zucchini or other summer squash (1/2 cup)
- ◯ Never
- ◯ Less than once per month
- ◯ 1–3 times per month
- ◯ Once per week
- ◯ 2–4 times per week
- ◯ 5–6 times per week
- ◯ 1 or more servings per day

Yams or sweet potatoes (1/2 cup)
- ◯ Never
- ◯ Less than once per month
- ◯ 1–3 times per month
- ◯ Once per week
- ◯ 2–4 times per week
- ◯ 5–6 times per week
- ◯ 1 or more servings per day

Spinach, cooked (1/2 cup)
- ◯ Never
- ◯ Less than once per month
- ◯ 1–3 times per month
- ◯ Once per week
- ◯ 2–4 times per week
- ◯ 5–6 times per week
- ◯ 1 or more servings per day

Spinach, raw as in salad (1 cup)
- ◯ Never
- ◯ Less than once per month
- ◯ 1–3 times per month
- ◯ Once per week
- ◯ 2–4 times per week
- ◯ 5–6 times per week
- ◯ 1 or more servings per day

Kale, mustard, or chard greens (1/2 cup)
- ◯ Never
- ◯ Less than once per month
- ◯ 1–3 times per month
- ◯ Once per week
- ◯ 2–4 times per week
- ◯ 5–6 times per week
- ◯ 1 or more servings per day

Iceberg or head lettuce (serving)
- ◯ Never
- ◯ Less than once per month
- ◯ 1–3 times per month
- ◯ Once per week
- ◯ 2–4 times per week
- ◯ 5–6 times per week
- ◯ Once per day
- ◯ 2 or more servings per day

Romaine or leaf lettuce (serving)
- ◯ Never
- ◯ Less than once per month
- ◯ 1–3 times per month
- ◯ Once per week
- ◯ 2–4 times per week
- ◯ 5–6 times per week
- ◯ Once per day
- ◯ 2 or more servings per day

PROOF

Celery (2–3 sticks)
- ◯ Never
- ◯ Less than once per month
- ◯ 1–3 per month
- ◯ Once per week
- ◯ 2–4 per week
- ◯ 5–6 per week
- ◯ Once per day
- ◯ 2 or more servings per day

Peppers: green, yellow or red (3 slices)
- ◯ Never
- ◯ Less than once per month
- ◯ 1–3 times per month
- ◯ Once per week
- ◯ 2–4 times per week
- ◯ 5–6 times per week
- ◯ 1 or more servings per day

Onions as a garnish or in a salad (1 slice)
- ◯ Never
- ◯ Less than once per month
- ◯ 1–3 slices per month
- ◯ 1 slice per week
- ◯ 2–4 slices per week
- ◯ 5–6 slices per week
- ◯ 1 or more slices per day

Onions as a vegetable, rings or soup (1/2 cup)
- ◯ Never
- ◯ Less than once per month
- ◯ 1–3 per month
- ◯ 1 per week
- ◯ 2–4 per week
- ◯ 5–6 per week
- ◯ 1 or more per day

3/8" spine perf

EGGS, MEAT & FISH

6. Please fill in your average total use, during the past year, of each specified food.

Egg Beaters or egg whites only (1/4 cup or 1 egg)
- Never
- Less than once per month
- 1–3 eggs per month
- 1 egg per week
- 2–4 eggs per week
- 5–6 eggs per week
- 1 egg per day
- 2 or more eggs per day

Omega-3 fortified eggs, including yolk (1 egg)
- Never
- Less than once per month
- 1–3 eggs per month
- 1 egg per week
- 2–4 eggs per week
- 5–6 eggs per week
- 1 egg per day
- 2 or more eggs per day

Regular eggs, with yolk (1)
- Never
- Less than once per month
- 1–3 eggs per month
- 1 egg per week
- 2–4 eggs per week
- 5–6 eggs per week
- 1 egg per day
- 2 or more eggs per day

Bacon (2 slices)
- Never
- Less than once per month
- 1–3 times per month
- Once per week
- 2–4 times per week
- 5–6 times per week
- 1 or more servings per day

Chicken or turkey sandwich or frozen dinner
- Never
- Less than once per month
- 1–3 times per month
- Once per week
- 2–4 times per week
- 5 or more per week

Other chicken or turkey, with skin (3 oz.)
- Never
- Less than once per month
- 1–3 times per month
- Once per week
- 2–4 times per week
- 5–6 times per week
- Once per day
- 2 or more servings per day

PROOF

Other chicken or turkey, including ground without skin, (3 oz.)
- Never
- Less than once per month
- 1–3 times per month
- Once per week
- 2–4 times per week
- 5–6 times per week
- Once per day
- 2 or more servings per day

Beef or pork hot dogs (1)
- Never
- Less than once per month
- 1–3 per month
- 1 per week
- 2–4 per week
- 5–6 per week
- 1 per day
- 2 or more per day

Chicken or turkey hot dogs or sausages (1)
- Never
- Less than once per month
- 1–3 per month
- 1 per week
- 2–4 per week
- 5–6 per week
- 1 per day
- 2 or more per day

Salami, bologna, or other processed meat sandwiches
- Never
- Less than once per month
- 1–3 times per month
- Once per week
- 2–4 times per week
- 5 or more per week

Other processed meats, e.g., sausage, kielbasa, etc. (2 oz. or 2 small links)
- Never
- Less than once per month
- 1–3 times per month
- Once per week
- 2–4 times per week
- 5–6 times per week
- Once per day
- 2 or more servings per day

Hamburger, lean or extra lean (1 patty)
- Never
- Less than once per month
- 1–3 per month
- 1 per week
- 2–4 per week
- 5–6 per week
- 1 or more per day

3/8" spine perf

6

6. (Continued) Please fill in your average total use, during the past year, of each specified food.

Hamburger, regular (1 patty)

- Never
- Less than once per month
- 1–3 per month
- 1 per week
- 2–4 per week
- 5–6 per week
- 1 or more per day

Beef, pork, or lamb as a sandwich or mixed dish, e.g., stew, casserole, lasagna, frozen dinner, etc.

- Never
- Less than once per month
- 1–3 times per month
- Once per week
- 2–4 times per week
- 5–6 times per week
- 1 or more times per day

Pork as a main dish, e.g., ham or chops (4–6 oz.)

- Never
- Less than once per month
- 1–3 times per month
- Once per week
- 2–4 times per week
- 5–6 times per week
- 1 or more times per day

Beef or lamb as a main dish, e.g., steak, roast (4–6 oz.)

- Never
- Less than once per month
- 1–3 times per month
- Once per week
- 2–4 times per week
- 5–6 times per week
- 1 or more times per day

Liver: beef, calf or pork (4 oz.)

- Never
- Less than once per month
- 1 time per month
- 2–3 times per month
- Once per week
- 2 or more servings per week

Liver: chicken or turkey (1 oz.)

- Never
- Less than once per month
- 1 time per month
- 2–3 times per month
- Once per week
- 2 or more servings per week

Canned tuna fish (3–4 oz.)

- Never
- Less than once per month
- 1–3 times per month
- Once per week
- 2–4 times per week
- 5–6 times per week
- Once per day
- 2 or more servings per day

Breaded fish cakes, pieces, or fish sticks (1 serving, store bought)

- Never
- Less than once per month
- 1–3 times per month
- Once per week
- 2–4 times per week
- 5–6 times per week
- 1 or more per day

Shrimp, lobster, scallops, clams as a main dish (1 serving)

- Never
- Less than once per month
- 1–3 times per month
- Once per week
- 2–4 times per week
- 5–6 times per week
- 1 or more times per day

Dark meat fish, e.g., tuna steak, mackerel, salmon, sardines, bluefish, swordfish (3–5 oz.)

- Never
- Less than once per month
- 1–3 times per month
- Once per week
- 2–4 times per week
- 5–6 times per week
- 1 or more servings per day

Other fish, e.g., cod, haddock, halibut (3–5 oz.)

- Never
- Less than once per month
- 1–3 times per month
- Once per week
- 2–4 times per week
- 5–6 times per week
- 1 or more servings per day

PROOF

3/8" spine perf

CEREALS, BREADS & STARCHES

7. Please fill in your average total use, during the past year, of each specified food.

Cold breakfast cereal (1 serving)
- ◯ Never
- ◯ Less than once per month
- ◯ 1–3 cups per month
- ◯ 1 cup per week
- ◯ 2–4 cups per week
- ◯ 5–6 cups per week
- ◯ 1 cup per day
- ◯ 2–3 cups per day
- ◯ 4 or more cups per day

Cooked oatmeal/cooked oat bran (1 cup)
- ◯ Never
- ◯ Less than once per month
- ◯ 1–3 cups per month
- ◯ 1 cup per week
- ◯ 2–4 cups per week
- ◯ 5–6 cups per week
- ◯ 1 cup per day
- ◯ 2–3 cups per day
- ◯ 4 or more cups per day

Other cooked breakfast cereal (1 cup)
- ◯ Never
- ◯ Less than once per month
- ◯ 1–3 cups per month
- ◯ 1 cup per week
- ◯ 2–4 cups per week
- ◯ 5–6 cups per week
- ◯ 1 cup per day
- ◯ 2–3 cups per day
- ◯ 4 or more cups per day

What brand and type of cold breakfast cereal do you usually eat? → Specify brand & type (e.g., "General Mills Rice Chex")

[ ]

	0	0	0
	1	1	1
Ch	2	2	2
rb	3	3	3
cf	4	4	4
sw	5	5	5
gn	6	6	6
t	7	7	7
k	8	8	8
w	9	9	9

0	0
1	1
2	2
3	3
4	4
5	5
6	6
7	7
8	8
9	9

PROOF

White bread (slice), including pita bread
- ◯ Never
- ◯ Less than once per month
- ◯ 1–3 slices per month
- ◯ 1 slice per week
- ◯ 2–4 slices per week
- ◯ 5–6 slices per week
- ◯ 1 slice per day
- ◯ 2–3 slices per day
- ◯ 4–5 slices per day
- ◯ 6+ slices per day

Rye or Pumpernickel bread (1 slice)
- ◯ Never
- ◯ Less than once per month
- ◯ 1–3 slices per month
- ◯ 1 slice per week
- ◯ 2–4 slices per week
- ◯ 5–6 slices per week
- ◯ 1 slice per day
- ◯ 2–3 slices per day
- ◯ 4–5 slices per day
- ◯ 6+ slices per day

Whole wheat, oatmeal, other whole grain bread (1 slice)
- ◯ Never
- ◯ Less than once per month
- ◯ 1–3 slices per month
- ◯ 1 slice per week
- ◯ 2–4 slices per week
- ◯ 5–6 slices per week
- ◯ 1 slice per day
- ◯ 2–3 slices per day
- ◯ 4–5 slices per day
- ◯ 6+ slices per day

Bagels, English muffins, or rolls (1 whole)
- ◯ Never
- ◯ Less than once per month
- ◯ 1–3 times per month
- ◯ Once per week
- ◯ 2–4 times per week
- ◯ 5–6 times per week
- ◯ Once per day
- ◯ 2 or more per day

Muffins or biscuits (1)
- ◯ Never
- ◯ Less than once per month
- ◯ 1–3 per month
- ◯ 1 per week
- ◯ 2–4 per week
- ◯ 5–6 per week
- ◯ 1 per day
- ◯ 2 or more per day

Brown rice (1 cup)
- ◯ Never
- ◯ Less than once per month
- ◯ 1–3 cups per month
- ◯ 1 cup per week
- ◯ 2–4 cups per week
- ◯ 5–6 cups per week
- ◯ 1 cup per day
- ◯ 2 or more cups per day

3/8" spine perf

7. (Continued) Please fill in your average total use, during the past year, of each specified food.

White rice (1 cup)

- ◯ Never
- ◯ Less than once per month
- ◯ 1–3 cups per month
- ◯ 1 cup per week
- ◯ 2–4 cups per week
- ◯ 5–6 cups per week
- ◯ 1 cup per day
- ◯ 2 or more cups per day

Pasta, e.g., spaghetti, noodles, couscous, etc. (1 cup)

- ◯ Never
- ◯ Less than once per month
- ◯ 1–3 cups per month
- ◯ 1 cup per week
- ◯ 2–4 cups per week
- ◯ 5–6 cups per week
- ◯ 1 cup per day
- ◯ 2 or more cups per day

Tortillas (2)

- ◯ Never
- ◯ Less than once per month
- ◯ 1–3 per month
- ◯ 1 per week
- ◯ 2–4 per week
- ◯ 5–6 per week
- ◯ 1 per day
- ◯ 2–3 per day
- ◯ 4 or more per day

Other grains, e.g., bulgar, kasha, buckwheat, etc. (1 cup)

- ◯ Never
- ◯ Less than once per month
- ◯ 1–3 cups per month
- ◯ 1 cup per week
- ◯ 2–4 cups per week
- ◯ 5–6 cups per week
- ◯ 1 cup per day
- ◯ 2 or more cups per day

Pancakes or waffles (2 small pieces)

- ◯ Never
- ◯ Less than once per month
- ◯ 1–3 servings per month
- ◯ 1 serving per week
- ◯ 2–4 servings per week
- ◯ 5–6 servings per week
- ◯ 1 serving per day
- ◯ 2 or more servings per day

French fried potatoes (6 oz. or 1 serving)

- ◯ Never
- ◯ Less than once per month
- ◯ 1–3 times per month
- ◯ Once per week
- ◯ 2–4 times per week
- ◯ 5–6 times per week
- ◯ 1 or more servings per day

Potatoes, baked, boiled (1) or mashed (1 cup)

- ◯ Never
- ◯ Less than once per month
- ◯ 1–3 per month
- ◯ 1 per week
- ◯ 2–4 per week
- ◯ 5–6 per week
- ◯ 1 per day
- ◯ 2 or more servings per day

Potato chips or corn/tortilla chips (small bag or 1 oz.)

- ◯ Never
- ◯ Less than once per month
- ◯ 1–3 per month
- ◯ 1 per week
- ◯ 2–4 per week
- ◯ 5–6 per week
- ◯ 1 per day
- ◯ 2 or more servings per day

Crackers, regular or low fat, e.g., Triscuits, Ritz (6)

- ◯ Never
- ◯ Less than once per month
- ◯ 1–3 times per month
- ◯ Once per week
- ◯ 2–4 times per week
- ◯ 5–6 times per week
- ◯ Once per day
- ◯ 2–3 times per day
- ◯ 4 or more servings per day

Pizza (2 slices)

- ◯ Never
- ◯ Less than once per month
- ◯ 1–3 times per month
- ◯ Once per week
- ◯ 2–4 times per week
- ◯ 5–6 times per week
- ◯ Once per day
- ◯ 2 or more servings per day

PROOF

3/8" spine perf

206915–5/5

BEVERAGES

CARBONATED BEVERAGES—Consider the serving size as one 12 oz. glass, bottle or can for these carbonated beverages.

8. Please fill in your average total use, during the past year, of each specified food.

LOW-CALORIE (Sugar-free types)

Low-calorie beverage with caffeine, e.g., Diet Coke, Diet Mt. Dew (1 glass, bottle or can)

- ◯ Never
- ◯ Less than once per month
- ◯ 1–3 cans per month
- ◯ 1 can per week
- ◯ 2–4 cans per week
- ◯ 5–6 cans per week
- ◯ 1 can per day
- ◯ 2–3 cans per day
- ◯ 4 or more cans per day

Other low-calorie beverage without caffeine, e.g., Diet 7-Up (1 glass, bottle or can)

- ◯ Never
- ◯ Less than once per month
- ◯ 1–3 cans per month
- ◯ 1 can per week
- ◯ 2–4 cans per week
- ◯ 5–6 cans per week
- ◯ 1 can per day
- ◯ 2–3 cans per day
- ◯ 4 or more cans per day

REGULAR TYPES (not sugar-free)

Carbonated beverage with caffeine and sugar, e.g., Coke, Pepsi, Mt. Dew, Dr. Pepper (1 glass, bottle or can)

- ◯ Never
- ◯ Less than once per month
- ◯ 1–3 cans per month
- ◯ 1 can per week
- ◯ 2–4 cans per week
- ◯ 5–6 cans per week
- ◯ 1 can per day
- ◯ 2–3 cans per day
- ◯ 4 or more cans per day

Other carbonated beverage with sugar, e.g., 7-Up, Root Beer, Ginger Ale (1 glass, bottle or can)

- ◯ Never
- ◯ Less than once per month
- ◯ 1–3 cans per month
- ◯ 1 can per week
- ◯ 2–4 cans per week
- ◯ 5–6 cans per week
- ◯ 1 can per day
- ◯ 2–3 cans per day
- ◯ 4 or more cans per day

PROOF

OTHER BEVERAGES

Other sugared beverages: Punch, lemonade, sports drinks, or sugared ice tea (1 glass, bottle, can)

- ◯ Never
- ◯ Less than once per month
- ◯ 1–3 glasses per month
- ◯ 1 glass per week
- ◯ 2–4 glasses per week
- ◯ 5–6 glasses per week
- ◯ 1 glass per day
- ◯ 2–3 glasses per day
- ◯ 4 or more glasses per day

Beer, regular (1 glass, bottle, can)

- ◯ Never
- ◯ Less than once per month
- ◯ 1–3 cans per month
- ◯ 1 can per week
- ◯ 2–4 cans per week
- ◯ 5–6 cans per week
- ◯ 1 can per day
- ◯ 2–3 cans per day
- ◯ 4–5 cans per day
- ◯ 6+ cans per day

Light beer, e.g., Bud Light (1 glass, bottle, can)

- ◯ Never
- ◯ Less than once per month
- ◯ 1–3 cans per month
- ◯ 1 can per week
- ◯ 2–4 cans per week
- ◯ 5–6 cans per week
- ◯ 1 can per day
- ◯ 2–3 cans per day
- ◯ 4–5 cans per day
- ◯ 6+ cans per day

3/8" spine perf

8

8. (Continued) Please fill in your average total use, during the past year, of each specified food.

Red wine (5 oz. glass)

- ◯ Never
- ◯ Less than once per month
- ◯ 1–3 glasses per month
- ◯ 1 glass per week
- ◯ 2–4 glasses per week
- ◯ 5–6 glasses per week
- ◯ 1 glass per day
- ◯ 2–3 glasses per day
- ◯ 4–5 glasses per day
- ◯ 6+ glasses per day

White wine (5 oz. glass)

- ◯ Never
- ◯ Less than once per month
- ◯ 1–3 glasses per month
- ◯ 1 glass per week
- ◯ 2–4 glasses per week
- ◯ 5–6 glasses per week
- ◯ 1 glass per day
- ◯ 2–3 glasses per day
- ◯ 4–5 glasses per day
- ◯ 6+ glasses per day

Liquor, e.g., whiskey, gin, etc. (1 drink or shot)

- ◯ Never
- ◯ Less than once per month
- ◯ 1–3 drinks per month
- ◯ 1 drink per week
- ◯ 2–4 drinks per week
- ◯ 5–6 drinks per week
- ◯ 1 drink per day
- ◯ 2–3 drinks per day
- ◯ 4–5 drinks per day
- ◯ 6+ drinks per day

Water, bottled, sparkling or tap (8 oz. cup)

- ◯ Never
- ◯ Less than once per month
- ◯ 1–3 cups per month
- ◯ 1 cup per week
- ◯ 2–4 cups per week
- ◯ 5–6 cups per week
- ◯ 1 cup per day
- ◯ 2–3 cups per day
- ◯ 4–5 cups per day
- ◯ 6+ cups per day

Herbal tea or decaffeinated tea (8 oz. cup)

- ◯ Never
- ◯ Less than once per month
- ◯ 1–3 cups per month
- ◯ 1 cup per week
- ◯ 2–4 cups per week
- ◯ 5–6 cups per week
- ◯ 1 cup per day
- ◯ 2–3 cups per day
- ◯ 4–5 cups per day
- ◯ 6+ cups per day

Tea with caffeine, including green tea (8 oz. cup)

- ◯ Never
- ◯ Less than once per month
- ◯ 1–3 cups per month
- ◯ 1 cup per week
- ◯ 2–4 cups per week
- ◯ 5–6 cups per week
- ◯ 1 cup per day
- ◯ 2–3 cups per day
- ◯ 4–5 cups per day
- ◯ 6+ cups per day

PROOF

Decaffeinated coffee (8 oz. cup)

- ◯ Never
- ◯ Less than once per month
- ◯ 1–3 cups per month
- ◯ 1 cup per week
- ◯ 2–4 cups per week
- ◯ 5–6 cups per week
- ◯ 1 cup per day
- ◯ 2–3 cups per day
- ◯ 4–5 cups per day
- ◯ 6+ cups per day

Coffee with caffeine (8 oz. cup)

- ◯ Never
- ◯ Less than once per month
- ◯ 1–3 cups per month
- ◯ 1 cup per week
- ◯ 2–4 cups per week
- ◯ 5–6 cups per week
- ◯ 1 cup per day
- ◯ 2–3 cups per day
- ◯ 4–5 cups per day
- ◯ 6+ cups per day

Dairy coffee drink (hot/cold) e.g., cappuccino (16 oz.)

- ◯ Never
- ◯ Less than once per month
- ◯ 1–3 cups per month
- ◯ 1 cup per week
- ◯ 2–4 cups per week
- ◯ 5–6 cups per week
- ◯ 1 cup per day
- ◯ 2–3 cups per day
- ◯ 4–5 cups per day
- ◯ 6+ cups per day

SWEETS, BAKED GOODS & MISCELLANEOUS

9. Please fill in your average total use, during the past year, of each specified food.

Milk chocolate (bar or packet), (e.g., Hershey's, M&M's)

- ◯ Never
- ◯ Less than once per month
- ◯ 1–3 per month
- ◯ 1 per week
- ◯ 2–4 per week
- ◯ 5–6 per week
- ◯ 1 per day
- ◯ 2–3 per day
- ◯ 4 or more per day

Dark chocolate e.g., Hershey's, Dark or Dove Dark

- ◯ Never
- ◯ Less than once per month
- ◯ 1–3 candy bars per month
- ◯ 1 candy bar per week
- ◯ 2–4 candy bars per week
- ◯ 5–6 candy bars per week
- ◯ 1 candy bar per day
- ◯ 2–3 candy bars per day
- ◯ 4 or more candy bars per day

Candy bars, (e.g., Snickers, Milky Way, Reeses)

- ◯ Never
- ◯ Less than once per month
- ◯ 1–3 candy bars per month
- ◯ 1 candy bar per week
- ◯ 2–4 candy bars per week
- ◯ 5–6 candy bars per week
- ◯ 1 candy bar per day
- ◯ 2–3 candy bars per day
- ◯ 4 or more candy bars per day

9

3/8" spine perf

206915–4/5

9. (Continued) Please fill in your average total use, during the past year, of each specified food.

Candy without chocolate (e.g., 1 pack mints, Lifesavers) (1 oz.)
- ◯ Never
- ◯ Less than once per month
- ◯ 1–3 times per month
- ◯ Once per week
- ◯ 2–4 times per week
- ◯ 5–6 times per week
- ◯ Once per day
- ◯ 2–3 times per day
- ◯ 4 or more times per day

Jams, jellies, preserves, syrup, or honey (1 tbs.)
- ◯ Never
- ◯ Less than once per month
- ◯ 1–3 tbs. per month
- ◯ 1 tbs. per week
- ◯ 2–4 tbs. per week
- ◯ 5–6 tbs. per week
- ◯ 1 tbs. per day
- ◯ 2–3 tbs. per day
- ◯ 4 or more tbs. per day

Peanut butter (1 tbs.)
- ◯ Never
- ◯ Less than once per month
- ◯ 1–3 tbs. per month
- ◯ 1 tbs. per week
- ◯ 2–4 tbs. per week
- ◯ 5–6 tbs. per week
- ◯ 1 tbs. per day
- ◯ 2–3 tbs. per day
- ◯ 4 or more tbs. per day

Fat free or light popcorn (3 cups)
- ◯ Never
- ◯ Less than once per month
- ◯ 1–3 servings per month
- ◯ 1 serving per week
- ◯ 2–4 servings per week
- ◯ 5–6 servings per week
- ◯ 1 serving per day
- ◯ 2 or more servings per day

Regular popcorn (3 cups)
- ◯ Never
- ◯ Less than once per month
- ◯ 1–3 servings per month
- ◯ 1 serving per week
- ◯ 2–4 servings per week
- ◯ 5–6 servings per week
- ◯ 1 serving per day
- ◯ 2 or more servings per day

Pretzels (1 small bag or serving)
- ◯ Never
- ◯ Less than once per month
- ◯ 1–3 servings per month
- ◯ One serving per week
- ◯ 2–4 servings per week
- ◯ 5–6 servings per week
- ◯ One serving per day
- ◯ 2 or more servings per day

Cookies, fat free or reduced fat (1)
- ◯ Never
- ◯ Less than once per month
- ◯ 1–3 cookies per month
- ◯ 1 cookie per week
- ◯ 2–4 cookies per week
- ◯ 5–6 cookies per week
- ◯ 1 cookie per day
- ◯ 2–3 cookies per day
- ◯ 4 or more cookies per day

Cookies, other ready made (1)
- ◯ Never
- ◯ Less than once per month
- ◯ 1–3 cookies per month
- ◯ 1 cookie per week
- ◯ 2–4 cookies per week
- ◯ 5–6 cookies per week
- ◯ 1 cookie per day
- ◯ 2–3 cookies per day
- ◯ 4 or more cookies per day

Cookies, home baked (1)
- ◯ Never
- ◯ Less than once per month
- ◯ 1–3 cookies per month
- ◯ 1 cookie per week
- ◯ 2–4 cookies per week
- ◯ 5–6 cookies per week
- ◯ 1 cookie per day
- ◯ 2–3 cookies per day
- ◯ 4 or more cookies per day

Brownies (1)
- ◯ Never
- ◯ Less than once per month
- ◯ 1–3 per month
- ◯ 1 per week
- ◯ 2–4 per week
- ◯ 5–6 per week
- ◯ 1 per day
- ◯ 2 or more per day

Doughnuts (1)
- ◯ Never
- ◯ Less than once per month
- ◯ 1–3 per month
- ◯ 1 per week
- ◯ 2–4 per week
- ◯ 5–6 per week
- ◯ 1 per day
- ◯ 2–3 per day
- ◯ 4 or more per day

Cake, home baked (slice)
- ◯ Never
- ◯ Less than once per month
- ◯ 1–3 slices per month
- ◯ 1 slice per week
- ◯ 2–4 slices per week
- ◯ 5–6 slices per week
- ◯ 1 or more slices per day

Cake, ready made (slice)
- ◯ Never
- ◯ Less than once per month
- ◯ 1–3 slices per month
- ◯ 1 slice per week
- ◯ 2–4 slices per week
- ◯ 5–6 slices per week
- ◯ 1 or more slices per day

Pie, homemade (slice)
- ◯ Never
- ◯ Less than once per month
- ◯ 1–3 slices per month
- ◯ 1 slice per week
- ◯ 2–4 slices per week
- ◯ 5–6 slices per week
- ◯ 1 or more slices per day

Pie, ready made (slice)
- ◯ Never
- ◯ Less than once per month
- ◯ 1–3 slices per month
- ◯ 1 slice per week
- ◯ 2–4 slices per week
- ◯ 5–6 slices per week
- ◯ 1 or more slices per day

PROOF
3/8" spine perf

9. (Continued) Please fill in your average total use, during the past year, of each specified food.

Sweet roll, coffee cake or other pastry, fat free or reduced fat (serving)
- ◯ Never
- ◯ Less than once per month
- ◯ 1–3 times per month
- ◯ Once per week
- ◯ 2–4 times per week
- ◯ 5–6 times per week
- ◯ Once per day
- ◯ 2 or more servings per day

Sweet roll, coffee cake or other ready made pastry (serving)
- ◯ Never
- ◯ Less than once per month
- ◯ 1–3 times per month
- ◯ Once per week
- ◯ 2–4 times per week
- ◯ 5–6 times per week
- ◯ Once per day
- ◯ 2 or more servings per day

Sweet roll, coffee cake or other pastry, home baked (serving)
- ◯ Never
- ◯ Less than once per month
- ◯ 1–3 times per month
- ◯ Once per week
- ◯ 2–4 times per week
- ◯ 5–6 times per week
- ◯ Once per day
- ◯ 2 or more servings per day

Peanuts (small packet or 1 oz.)
- ◯ Never
- ◯ Less than once per month
- ◯ 1–3 per month
- ◯ 1 per week
- ◯ 2–4 per week
- ◯ 5–6 per week
- ◯ 1 per day
- ◯ 2 or more servings per day

Walnuts (1 oz.)
- ◯ Never
- ◯ Less than once per month
- ◯ 1–3 per month
- ◯ 1 per week
- ◯ 2–4 per week
- ◯ 5–6 per week
- ◯ 1 per day
- ◯ 2 or more servings per day

Other nuts (small packet or 1 oz.)
- ◯ Never
- ◯ Less than once per month
- ◯ 1–3 per month
- ◯ 1 per week
- ◯ 2–4 per week
- ◯ 5–6 per week
- ◯ 1 per day
- ◯ 2 or more servings per day

Breakfast bars, e.g., Nutrigrain, granola, Kashi (1)
- ◯ Never
- ◯ Less than once per month
- ◯ 1–3 per month
- ◯ 1 per week
- ◯ 2–4 per week
- ◯ 5–6 per week
- ◯ 1 per day
- ◯ 2 or more bars per day

Energy bars, e.g., Clif, Luna, Glucerna, Powerbar (1)
- ◯ Never
- ◯ Less than once per month
- ◯ 1–3 per month
- ◯ 1 per week
- ◯ 2–4 per week
- ◯ 5–6 per week
- ◯ 1 per day
- ◯ 2 or more bars per day

Low carb bars, e.g., Atkins, Zone, South Beach (1)
- ◯ Never
- ◯ Less than once per month
- ◯ 1–3 per month
- ◯ 1 per week
- ◯ 2–4 per week
- ◯ 5–6 per week
- ◯ 1 per day
- ◯ 2 or more bars per day

Oat bran, added to food (1 tbs.)
- ◯ Never
- ◯ Less than once per month
- ◯ 1–3 tbs. per month
- ◯ 1 tbs. per week
- ◯ 2–4 tbs. per week
- ◯ 5–6 tbs. per week
- ◯ 1 tbs. per day
- ◯ 2 or more servings per day

Other bran (wheat, etc.), added to food (1 tbs.)
- ◯ Never
- ◯ Less than once per month
- ◯ 1–3 tbs. per month
- ◯ 1 tbs. per week
- ◯ 2–4 tbs. per week
- ◯ 5–6 tbs. per week
- ◯ 1 tbs. per day
- ◯ 2 or more servings per day

Wheat germ (1 tbs.)
- ◯ Never
- ◯ Less than once per month
- ◯ 1–3 tbs. per month
- ◯ 1 tbs. per week
- ◯ 2–4 tbs. per week
- ◯ 5–6 tbs. per week
- ◯ 1 tbs. per day
- ◯ 2 or more servings per day

Chowder or cream soup (1 cup)
- ◯ Never
- ◯ Less than once per month
- ◯ 1–3 cups per month
- ◯ 1 cup per week
- ◯ 2–4 cups per week
- ◯ 5–6 cups per week
- ◯ 1 or more cups per day

Ketchup or red chili sauce (1 tbs.)
- ◯ Never
- ◯ Less than once per month
- ◯ 1–3 tbs. per month
- ◯ 1 tbs. per week
- ◯ 2–4 tbs. per week
- ◯ 5–6 tbs. per week
- ◯ 1 tbs. per day
- ◯ 2 or more servings per day

PROOF

3/8" spine perf

206915–3/5

9. (Continued) Please fill in your average total use, during the past year, of each specified food.

Salt added at table (1 shake)
- Never
- Less than once per month
- 1–3 shakes per month
- 1 shake per week
- 2–4 shakes per week
- 5–6 shakes per week
- 1 shake per day
- 2–3 shakes per day
- 4–5 shakes per day
- 6+ shakes per day

How many teaspoons of sugar do you add to your beverages or food each day?

Teaspoons

0 1 2 3 4 5 6 7 8 9
0 1 2 3 4 5 6 7 8 9

Splenda (1 packet)
- Never
- Less than once per month
- 1–3 per month
- 1 per week
- 2–4 per week
- 5–6 per week
- 1 per day
- 2–3 per day
- 4–5 per day
- 6+ per day

Other artificial sweetener (1 packet)
- Never
- Less than once per month
- 1–3 per month
- 1 per week
- 2–4 per week
- 5–6 per week
- 1 per day
- 2–3 per day
- 4–5 per day
- 6+ per day

Garlic (1 clove or 4 shakes)
- Never
- Less than once per month
- 1–3 per month
- 1 per week
- 2–4 per week
- 5–6 per week
- 1 per day
- 2–3 per day
- 4–5 per day
- 6+ per day

Low fat or fat free mayonnaise (1 tbs.)
- Never
- Less than once per month
- 1–3 tbs. per month
- 1 tbs. per week
- 2–4 tbs. per week
- 5–6 tbs. per week
- 1 tbs. per day
- 2 or more tbs. per day

Regular mayonnaise (1 tbs.)
- Never
- Less than once per month
- 1–3 tbs. per month
- 1 tbs. per week
- 2–4 tbs. per week
- 5–6 tbs. per week
- 1 tbs. per day
- 2 or more tbs. per day

Salad dressing (1–2 tbs.) →
- Never
- Less than once per month
- 1–3 tbs. per month
- 1 tbs. per week
- 2–4 tbs. per week
- 5–6 tbs. per week
- 1 tbs. per day
- 2–3 tbs. per day
- 4 or more tbs. per day

Type of salad dressing:
- Nonfat
- Low fat
- Olive oil dressing
- Other vegetable oil dressing

Olive oil added to food or bread (1 tbs.); exclude use in cooking
- Never
- Less than once per month
- 1–3 tbs. per month
- 1 tbs. per week
- 2–4 tbs. per week
- 5–6 tbs. per week
- 1 tbs. per day
- 2–3 tbs. per day
- 4–5 tbs. per day
- 6+ tbs. per day

PROOF

3/8" spine perf

10. What kind of fat is usually used for frying and sautéing at home? (Exclude Pam type spray.)

- ◯ Real butter
- ◯ Margarine
- ◯ Olive oil
- ◯ Vegetable oil
- ◯ Vegetable shortening
- ◯ Lard/bacon fat

11. What kind of fat is usually used for baking at home? (Exclude Pam type spray.)

- ◯ Real butter
- ◯ Margarine
- ◯ Olive oil
- ◯ Vegetable oil
- ◯ Vegetable shortening
- ◯ Lard/bacon fat

12. How often do you eat food fried, stir-fried in oil, or sautéed at home?

- ◯ Never
- ◯ Less than once a week
- ◯ 1–3 times per week
- ◯ 4–6 times per week
- ◯ Daily

13. How often do you eat deep fried food away from home or as take out (e.g., french fries, fried chicken, fish, clams, shrimp, etc.)?

- ◯ Never
- ◯ Less than once a week
- ◯ 1–3 times per week
- ◯ 4–6 times per week
- ◯ Daily
- ◯ 2 or more times per day

14. How often do you eat toasted breads, bagel or English muffin (e.g., sliced/half bagel)?

- ◯ Never
- ◯ Less than once a week
- ◯ 1–3 times per week
- ◯ 4–6 times per week
- ◯ Daily
- ◯ 2 or more times per day

15. What type of cooking oil is usually used at home (e.g., Mazola Corn Oil)?

(Specify brand and type)

OLV CAN CRN SOY VEG

16. Are there any other foods not mentioned above that you usually eat at least once per week?

Include for example: Mushrooms, radish, horseradish, dates, figs, rhubarb, mango, mixed dried fruit, papaya, custard, venison, hot peppers, pickles, olives, SlimFast, Ensure (regular or plus), Glucerna shake.

(Do not include dry spices and do not list something that has been listed in the previous sections.)

Other foods that you usually eat at least once per week	Servings per week
(a)	
(b)	
(c)	

mus rad hrd dat fig rhu man mdf pap cus ven htp pic olv slm en en+ gls

PROOF

3/8" spine perf

DIET CHANGES

17. Do you currently follow a special diet?

- ◯ No
- ◯ Yes → ◯ Physician prescribed ◯ Self prescribed

a) If yes, for how many years?

(Number of years on diet)

[ ]

b) If yes, what kind of diet do you follow? (Select more than one if necessary.)

- ◯ Weight reduction (low calorie)
- ◯ Low cholesterol
- ◯ Low sodium
- ◯ Diabetic
- ◯ Low fat
- ◯ Low triglyceride
- ◯ Ulcer
- ◯ High Potassium
- ◯ Other → (Specify type of diet) (Exclude weight reduction diets.) [ ]

PROOF

18. How has your use of the following foods and beverages changed over the PAST TEN YEARS?

Whole milk
- ◯ Use has decreased
- ◯ Use about the same
- ◯ Use has increased

Butter
- ◯ Use has decreased
- ◯ Use about the same
- ◯ Use has increased

Margarine
- ◯ Use has decreased
- ◯ Use about the same
- ◯ Use has increased

Eggs
- ◯ Use has decreased
- ◯ Use about the same
- ◯ Use has increased

Fish
- ◯ Use has decreased
- ◯ Use about the same
- ◯ Use has increased

Red meat
- ◯ Use has decreased
- ◯ Use about the same
- ◯ Use has increased

Fruits
- ◯ Use has decreased
- ◯ Use about the same
- ◯ Use has increased

Vegetables
- ◯ Use has decreased
- ◯ Use about the same
- ◯ Use has increased

Whole wheat bread
- ◯ Use has decreased
- ◯ Use about the same
- ◯ Use has increased

Whole grains
- ◯ Use has decreased
- ◯ Use about the same
- ◯ Use has increased

Sugar
- ◯ Use has decreased
- ◯ Use about the same
- ◯ Use has increased

Alcohol
- ◯ Use has decreased
- ◯ Use about the same
- ◯ Use has increased

3/8" spine perf

Thank you!

PROOF

Please check to make sure you have not accidentally skipped any pages.

3/8" spine perf

Mark Reflex® forms by NCS Pearson EM-206915-2:654321 Printed in U.S.A.
Copyright © 2007 President and Fellows of Harvard College. All Rights Reserved Worldwide.

PLEASE DO NOT WRITE IN THIS AREA

SERIAL #
